# Uracil–DNA glycosylase UNG1 isoform variant supports class switch recombination and repairs nuclear genomic uracil

**DOI:** 10.1093/nar/gkz145

**Published:** 2019-03-06

**Authors:** Antonio Sarno, Marie Lundbæk, Nina Beate Liabakk, Per Arne Aas, Robin Mjelle, Lars Hagen, Mirta M L Sousa, Hans E Krokan, Bodil Kavli

**Affiliations:** 1 Department of Clinical and Molecular Medicine, NTNU-Norwegian University of Science and Technology, NO-7491 Trondheim, Norway; 2 Clinic of Laboratory Medicine, St. Olav's Hospital, Trondheim University Hospital, NO-7006 Trondheim, Norway; 3 PROMEC Core Facility for Proteomics and Modomics at NTNU and the Central Norway Regional Health Authority

## Abstract

UNG is the major uracil-DNA glycosylase in mammalian cells and is involved in both error-free base excision repair of genomic uracil and mutagenic uracil-processing at the antibody genes. However, the regulation of UNG in these different processes is currently not well understood. The *UNG* gene encodes two isoforms, UNG1 and UNG2, each possessing unique N-termini that mediate translocation to the mitochondria and the nucleus, respectively. A strict subcellular localization of each isoform has been widely accepted despite a lack of models to study them individually. To determine the roles of each isoform, we generated and characterized several UNG isoform-specific mouse and human cell lines. We identified a distinct UNG1 isoform variant that is targeted to the cell nucleus where it supports antibody class switching and repairs genomic uracil. We propose that the nuclear UNG1 variant, which in contrast to UNG2 lacks a PCNA-binding motif, may be specialized to act on ssDNA through its ability to bind RPA. RPA-coated ssDNA regions include both transcribed antibody genes that are targets for deamination by AID and regions in front of the moving replication forks. Our findings provide new insights into the function of UNG isoforms in adaptive immunity and DNA repair.

## INTRODUCTION

Uracil is a canonical RNA base that is also present at low levels in DNA. Genomic uracil is the result of replicative incorporation of dUMP instead of dTMP (resulting in U:A pairs) and spontaneous or enzymatic deamination of cytosine (resulting in U:G mispairs) (1,2). In mammalian cells, cytosine can be deaminated by the AID/APOBEC family of cytidine deaminases (3). AID deaminates cytosine in specific regions of the immunoglobulin (Ig) genes, as the initial step of the adaptive antibody affinity maturation processes - class switch recombination (CSR) and somatic hyper mutation (SHM) (4). Similarly, several APOBECs deaminate viral DNA as part of the innate immune response to combat virus infection (5,6). Importantly, untargeted activities of the AID/APOBEC deaminases are associated with mutagenesis in multiple human cancers (7,8), suggesting an important role for genomic uracil in cancer development.

Uracil in the genome is usually processed by a uracil-DNA glycosylase (UDG) that initiates the base excision repair (BER) pathway. Mammalian cells express several UDG enzymes (UNG, SMUG1, TDG and MBD4). UNG is responsible for most of the DNA uracil-excision activity in proliferating cells (9,10). In addition to its role in BER, studies on UNG-knockout mice and human patients with inactivating mutations in the *UNG* gene have demonstrated an essential role of UNG in adaptive immunity. UNG is required for CSR and modulates the SHM mutational pattern by processing AID-induced uracil (U:G) at the Ig genes (11,12).

**Figure 1.**
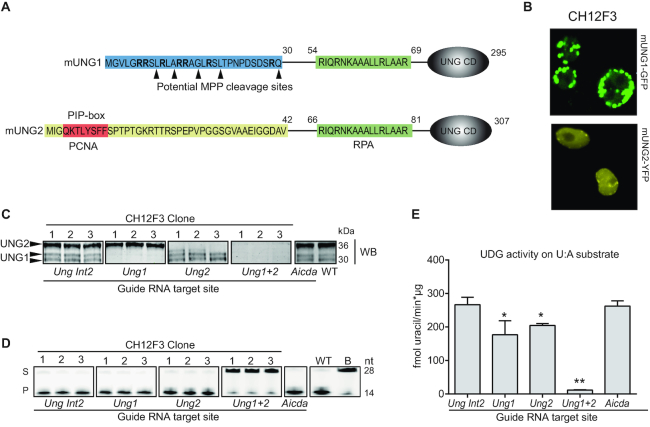
Generation and verification of UNG1 and UNG2 isoform-specific knockout clones in the mouse B-cell line CH12F3. (**A**) N-terminal amino acid sequence of mouse UNG1 and UNG2. UNG1-specific residues (amino acids 1–30) that target UNG1 to mitochondria are marked in blue. Arginine (R) residues in bold and potential target sites for proteolytic processing by MPP (mitochondrial processing peptidase) are indicated. UNG2-specific residues (amino acids 1–42) essential for nuclear localization are marked in yellow and include the PCNA-interacting peptide sequence (PIP-box in red) that targets UNG2 to the replisome. UNG1 and UNG2 both contain binding sites for RPA (green). UNG CD indicates the globular catalytic domain, which is present in both UNG1 and UNG2. (**B**) Confocal images of live stably transfected CH12F3 cells expressing tetracycline-inducible mUNG1-GFP or mUNG2-YFP. Cells were analyzed ∼24 hours post induction. (**C**) CH12F3 CRISPR/Cas9 sub-clones screened by western blot to detect UNG protein isoforms. Three independent clones representing each knockout are shown. *Ung Int2* clones, generated using an RNA guide with target sites in intron 2 of the *Ung* gene, are used as controls. (**D**) CH12F3 CRISPR/Cas9 sub-clones screened by UDG activity assay on whole cell extracts using a FAM-labeled 28 nucleotide (nt) ssDNA oligo with a central uracil as substrate (S). Uracil excision activity is demonstrated by the formation of a 14 nt product (P). (**E**) UDG activity assay using high molecular weight ^3^H-U:A nick-translated DNA as substrate. The bars represent mean activity of three independent clones in each group. Significantly reduced UDG activity compared to WT (Ung Int2) is indicated with *P< 0.05 and **P< 0.005.

The use of separate promoters and alternative splicing give rise to two different UNG-coding mRNA transcripts (13). The resulting isoforms, UNG1 and UNG2, have different N-terminal sequences but share the globular catalytic domain (14) and the binding motif for the nuclear ssDNA-binding protein RPA (15,16) (Figure 1A). The current paradigm is that UNG1 is transported to mitochondria where it is processed at the N-terminus by the mitochondrial processing peptidase (MPP) (17,18), while the UNG2 isoform is targeted to the nucleus. UNG2 can interact with PCNA by its N-terminal PIP-box motif (Figure 1) (19) or with RPA, to remove uracil at the replication fork (20). In addition, UNG2 is generally believed to be the isoform involved in CSR and SHM (4).

Although cells from mice and human patients completely deficient in UNG have been characterized, cell models to study the roles of the UNG1 and UNG2 isoforms separately have not previously been available. Here, we have generated and characterized mouse and human UNG isoform-specific knockout cell lines and investigated the regulation, cellular localization and function of endogenously expressed UNG1 and UNG2 separately. We demonstrate that a specific UNG1 variant is targeted to the cell nucleus where it supports Ig class switching and genomic uracil repair. We propose that this nuclear UNG1 variant interacts with RPA to preferentially process uracil in ssDNA regions.

## MATERIALS AND METHODS

### Cell lines and primary cells

All cell lines were cultured at 37 °C and 5% CO_2_ in media supplemented with 2 mM l-glutamine (Sigma), 10% fetal calf serum (FCS) (Sigma), 1× PenStrep solution (Gibco). CH12F3 cells (mouse B lymphocytes, class switch-proficient) were cultured in RPMI-1640 (Sigma) supplemented with heat-inactivated (56 °C, 30 min) FCS and 50 μM 2-mercaptoethanol (Gibco). CH12F3 cells were stimulated with 2 μg/ml hamster anti-mouse CD40 (BD Biosciences), 10 ng/ml recombinant murine IL-4 (Peprotech) and 1 ng/ml human recombinant TGF-β1 (PeproTech). L428 (human B lymphocytes, DSMZ ACC 197) and JC (mouse epithelial, ATCC CRL-2116™) cells were cultured in RPMI-1640 with heat-inactivated FCS and 1.25 μg/ml Amphotericin B (Sigma). U2OS (human osteosarcoma, ATCC HTB-96™), HeLa S3 (human epithelial, ATCC CCL-2.2™), HEK293T (virus packaging human cell line, Open Biosystems), CMT-93 (mouse epithelial, ATCC CCL-223™) and NIH-3T3 (mouse fibroblast, ATCC CRL-1658™) were cultured in DMEM–high glucose (Sigma) and 1.25 μg/ml Amphotericin B. Hap1 cells (Horizon) were cultured in IMDM (Gibco). U373 (human glioblastoma, ATCC HTB-17) were cultured in EMEM (Sigma) supplemented with 1× Non-Essential Amino Acids (Sigma) and 1 mM sodium pyruvate. Naïve resting B cells were isolated from spleens from three 9–10 month old WT mice (strain C57BL/6J) using EasySep Mouse B cell Isolation kit (StemCell) according to the manufacturers’ instructions. The isolated B cells were cultured in RPMI-1640 supplemented with 2 mM L-glutamine, 10% heat-inactivated FCS, 1 mM sodium pyruvate, 50 μM 2-mercaptoethanol and 1× PenStrep and stimulated with 40 μg/ml LPS (from *E. coli* strain 0111:B4, Merck) and 20 ng/ml IL-4 (PeproTech).

### Antibodies for western analysis

Primary antibodies: Monoclonal rat anti-AID (Active Motif, 39886); Polyclonal rabbit anti-mouse UNG (UNG 6103, custom made); Rabbit anti-human UNG (UNG PU059, made in-house (21)); Monoclonal rabbit anti-MRPL11 (D68F2) XP (Cell Signaling 2066); Monoclonal mouse anti-β-Actin (ab8226); Polyclonal rabbit anti-GFP (ab290); Monoclonal rabbit anti-RPA2 [EPR2877Y] (ab76420): Polyclonal rabbit anti PCNA Ab (ab18197). Secondary antibodies: HRP-conjugated swine anti-rabbit IgG (Dako); HRP-conjugated goat anti-mouse IgG (Dako); HRP-conjugated goat anti-rat IgG (Cell Signaling); IRDye 800CW Goat anti-rabbit (LI-COR); IRDye 680RD goat anti-rabbit (Li-COR); IRDye 680RD goat anti-mouse (Li-COR).

### Whole cell-, nuclear- and mitochondrial protein extracts

Whole cell extracts were prepared by suspending cell pellets in one cell pellet volume of buffer I (10 mM Tris–HCl pH 8, 200 mM KCl) before addition of one cell suspension volume of buffer II (10 mM Tris–HCl pH 8, 200 mM KCl, 2 mM EDTA, 40% glycerol, 0.5% NP-40, 2 mM DTT, 2x Complete^®^ protease inhibitor (Roche), 2× PIC (phosphatase inhibitor cocktails II and III, Sigma), 2× Omnicleave™ endonuclease, 10 μg/ml RNaseA, (Cambio), and 10 mM MgCl_2_). The mixture was rocked at 4°C for 1 h. Cell debris was removed by high speed centrifugation.

Nuclear extracts were prepared by washing the cells in 10 cell pellet volumes of isotonic buffer (20 mM HEPES pH 7.8, 1 mM MgCl_2_, 5 mM KCl, 250 mM sucrose, 1 mM DTT and 1× Complete^®^). Cells were suspended in five cell-pellet volumes of hypotonic buffer (20 mM HEPES pH 7.8, 1 mM MgCl_2_, 5 mM KCl, 1 mM DTT and 1× Complete^®^) and incubated for 10 min on ice for swelling before lysis in a Dounce homogenizer using ten strokes of the pestle (type B, tight fit). Nuclei were isolated by centrifugation at 600 x *g* for 6 min. Pellets were suspended in two cell pellet volumes of hypertonic extraction buffer (20 mM HEPES pH 7.8, 1mM MgCl_2_, 5mM KCl, 500 mM NaCl, 25% glycerol, 1mM DTT and 1x Complete^®^) and proteins were extracted by rocking at 4 °C for 30 min. The nuclear extracts were clarified by centrifugation at high speed.

Mitochondrial extracts were prepared from ∼10^6^ cells. Cells were suspended in 1 ml MSHE (10 mM HEPES pH 7.4, 70 mM sucrose, 210 mM mannitol, 1 mM EDTA, 1 mM EGTA, 1XPIC, 1 mM DTT). The cell suspension were snap frozen in liquid nitrogen, thawed on ice and homogenized by 30 pestle strokes (type B, tight fit). Nuclei and cell debris were removed by centrifugation at 500 x *g* for 10 min. Mitochondria were separated from cytosolic proteins by centrifugation at 9500 x *g* for 10 min and treated with proteinase K (60 μg at 20 °C and 50–200 μg at 37 °C, CH12F3 and Transfected NIH-3T3, respectively) in 1 ml MSHE for 30 min to remove nuclear and cytosolic contaminants. Proteolytic cleavage was stopped by adding 0.2 mM PMSF and mitochondria were collected by centrifugation (9500 x *g*, 10 min). Mitochondrial proteins were extracted in lysis buffer (20 mM HEPES pH 7.4, 300 mM KCl, 0.5% Triton-X-100, 1 mM EDTA, 1 mM EGTA, 0.5 mM PMSF, 1XPIC, 1 mM DTT) on ice for 5 min. Debris were removed by high-speed centrifugation.

All extracts were snap frozen in liquid nitrogen and stored at −80 °C. Protein concentrations were measured using the Bradford assay (BioRad).

### Generation of CRISPR/Cas9 knockout clones

Single guide RNAs (sgRNAs) were designed using the CRISPR design tool from the F. Zhang laboratory (http://crispr.mit.edu/). To generate UNG isoform-specific knockout (KO) clones, we designed sgRNAs targeting the unique exon sequences of UNG1 and UNG2. To prepare UNG1+2 KO clones, we targeted the common sequence of exon 1B (13,22). sgRNA recognizing Intron 2 was designed to generate UNG-proficient control clones. Finally, we generated CSR-deficient CH12F3 clones by targeting exon 2 of the *AID* (*Aicda*) gene. The sequences of the single guide DNA oligonucleotides (Sigma) used are listed in Table 1. When preparing CH12F3, L428 and U373 knockout clones, the DNA oligonucleotides (Table 1) were annealed and cloned into the Cas9-expressing lentiCRISPRv2 vector (Addgene; plasmid # 52961) as described in the Zhang lab protocol (23,24). HEK293T cells were co-transfected with translentiviral packaging mix (TLP4606, Thermo Scientific) and lentiCRISPRv2 vectors expressing Cas9 and sgRNAs using X-tremeGENE HP transfection agent (Roche). Supernatants containing lentiviral particles were collected after 24, 48 and 72 h, sterile filtered, supplemented with protamine sulfate (10 μg/ml), and added to the target cell lines (CH12F3 and L428). Transduced cells were subsequently selected with puromycin (1 μg/ml) and sub-cloned by dilution. When generating Hap1 knockout cells, the guide DNA oligoes were cloned into the Cas9- and GFP-expressing PX458 gRNA vector (Addgene, plasmid # 48138) and cells were transfected using Viromer Red (Lipocalyx) transfection regents. GFP positive cells were sorted 48 h post transfection on a FACS Aria cell sorter. Cells were cultured further for 7–10 days before subcloning by single-cell sorting into a 96-well plates. Subclones were subsequently screened by western blot analysis. All clones were negative for mycoplasma as tested by DNA sequencing.

**Table 1.** Single guide oligo sequences for cloning and CRISPR/Cas9 targeting.

Guide ID	Species	Target gene and region	DNA oligo sequence	For/Rev
mUng1_4	Mouse	*Ung1*-specific region of exon 1B	caccgGCTGGACCATGGGCGTCTTG	+
			aaacCAAGACGCCCATGGTCCAGCc	-
mUng1_5	Mouse	*Ung1*-specific region of exon 1B	caccgTTGCGGTTGGCGCGGAGAGC	+
			aaacGCTCTCCGCGCCAACCGCAAc	-
mUng2_1	Mouse	*Ung2*–specific exon 1A	caccgCACGGCATCGCCGCCGATCT	+
			aaacAGATCGGCGGCGATGCCGTGc	-
mUng1+2	Mouse	*Ung* (common region, exon 1B)	caccgCTGCCGGCTTCGGCGAGAGC	+
			aaacGCTCTCGCCGAAGCCGGCAGc	-
mUng_int2	Mouse	*Ung* (intron 2)	caccgGTTAAATCAGGTTGGCGGGC	+
			aaacGCCCGCCAACCTGATTTAACc	-
mAid_ex2	Mouse	*Aicda* (exon 2)	caccgGTAGGTCTCATGCCGTCCCT	+
			aaacAGGGACGGCATGAGACCTACc	-
hUNG1	Human	*UNG1*-specific region, exon 1B	CaccgGCCGGAAGCTGCGGACGCCT	+
			aaacAGGCGTCCGCAGCTTCCGGCc	-
hUNG2	Human	*UNG2-*specific region, exon 1A	caccgCGTCTTCTGGCCGATCATCC	+
			aaacGGATGATCGGCCAGAAGACGc	-
hUNG1+2	Human	*UNG* (common region, exon 1B)	caccgGCGGCCCGCAACGTGCCCGT	+
			aaacACGGGCACGTTGCGGGCCGCc	-
hUNG_int2	Human	*UNG* (intron 2)	caccgCGACCCGCGAGATGATATCA	+
			aaacTGATATCATCTCGCGGGTCGc	-

### Sequence verification of targeted genome editing events

Genomic DNA was isolated from cell pellets using the DNeasy Blood & Tissue kit (Qiagen). The target loci of potential knockout clones were amplified and tagged with 6mer barcodes in both directions in nested PCR reactions. The resulting amplicons (∼200 bp) from the different clones were subsequently pooled and sent to GATC Biotech AG in Germany for multiplexed amplicon sequencing (Illumina HiSeq 4000, 125 bp PE/150 bp PE). The sequencing data were analyzed in-house implementing CRISPResso (http://crispresso.rocks/), a computational pipeline for the analysis of CRISPR-Cas9 genome editing outcomes from deep sequencing data developed by Pinello and colleagues (25). The unique combinations of forward and reverse 6mer barcodes were used to demultiplex the sequencing data.

### UDG activity assays

Standard UDG activity assays were performed in 20 μl reaction mixtures containing 1.8 μM nick translated [^3^H]-dUMP-labeled calf thymus DNA (U:A substrate), 2 μg whole cell protein extract, and 1x UDG buffer (20 mM Tris–HCl pH 7.5, 60 mM NaCl, 1 mM EDTA, 1 mM DTT, 0.5 mg/ml BSA) were incubated for 10 minutes at 30 °C. Acid-soluble [^3^H] uracil was quantified by scintillation counting as described (26).

Oligonucleotide UDG activity assays were measured using 10 μl mixtures containing 0.1 μM 6-FAM-labeled single stranded oligonucleotide (*T_12_-AG**U**A-T_12_) substrate, 1× UDG buffer and 2 μg whole cell protein extract. The assay mixtures were incubated at 30 °C for 30 min. Reactions were stopped and AP-sites were cleaved by addition of 50 μl 10% piperidine and incubation at 90 °C for 20 min. Product (14 nucleotides) and substrate (28 nucleotides) bands were separated by PAGE and visualized on Typhoon Trio imager (GE Healthcare).

### Cell synchronization, cell cycle analysis and expression of UNG isoforms during the cell cycle

CH12F3 cells (2×10^5^/ml, 3×10^6^/culture dish) of the various genotypes were seeded and cultured for 24 h before addition of 10 μM of the CDK1/cyclin B1 inhibitor RO-3306 (Sigma, SML0569-5MG), which arrest cells in G2/M. The cells were incubated with the inhibitor for 20 h before they were released. Cells were then harvested at several time points and fixed in 70% methanol, washed twice with PBS, and then treated with RNaseA (100 μg/ml in PBS) at 37 °C for 30 min prior to DNA staining with propidium iodide (50 μg/ml in PBS, 37 °C for 30 min). Cell cycle experiments were run on a FACS Canto flow cytometer (BD-Life Science) and analyzed using the FlowJo^®^ version 10 software package. UNG protein expression was analyzed by western blots on total cell extracts, and quantified relative to β-actin by the Kodak Molecular Imaging NE4 software.

### Class switch recombination assay

*In vitro* IgM to IgA class switching was measured using flow cytometry. CH12F3 cells (10 000 cells/ml) were seeded in flat-bottomed 96-well culture dishes in 200 μl growth medium. Cells were stimulated with 2 μg/ml hamster anti-mouse CD40 (BD Biosciences) and 10 ng/ml recombinant murine IL-4 (Peprotech), and 1 ng/ml human recombinant TGF-β1 (PeproTech) for 4 days. The cells were then stained with LIVE/DEAD red stain (Invitrogen), blocked with Fc receptor antibody (2.4G2) and normal mouse serum (Invitrogen), fixed and permeabilized in CytoFix/CytopermTM and washed in PermWashTM containing saponin. IgA was stained using anti-mouse IgA-PE (eBioscience, 1:200). Cells were washed twice with PermWashTM and suspended in 200 μl of CellFixTM before analysis on a FACS Canto. Viable CH12F3 cells were analyzed for IgA expression using FlowJo® version 10 software. Reagents were from BD Biosciences if not stated otherwise.

### Quantification of proteins by targeted mass spectrometry

Whole cell extracts (50 μg total protein) prepared from CH12F3 cells at several time points after seeding/stimulation were incubated in 5 mM tris(2-carboxyethyl) phosphine (TCEP) for 30 min at room temperature followed by alkylation with iodoacetamide (1 μmol/mg protein) for 30 min in the dark. Proteins were precipitated using a methanol-chloroform method as described (27) and subjected to another round of protein reduction and alkylation by resuspension and incubation for 30 min in 50 mM NH_4_HCO_3_ and 5 mM TCEP, before incubation with iodoacetamide. Trypsin (Thermo Scientific, Waltham, MA) was added at 1:50 ratio prior to overnight digestion at 37 °C in a shaker. Subsequently, 0.1% formic acid was added followed by centrifugation (10 min, 16 000 g) for removal of insoluble particles prior to mass spectrometry analysis. All parallel reaction monitoring (PRM)-based targeted mass spectrometry methods were designed, analyzed, and processed using Skyline software version 3.6.0.10162 (28). In silico selection of proteotypic peptides was performed via Skyline using the Mus musculus reference proteome available at www.uniprot.org to exclude non-unique peptides.

Peptides generated from tryptic digestion of recombinant AID and UNG proteins (1 μg) were used as standards and analyzed on a Thermo Scientific Q Exactive mass spectrometer operating in Targeted-MS2 mode. Information on their retention time and fragmentation pattern was used for peptide identification and to build a method with a retention time window of 4 min. The method was then employed for detection and quantification of corresponding peptides in murine samples.

Tryptic digested murine samples were analyzed on a Q Exactive mass spectrometer operating in Targeted-MS2 mode coupled to an EASY-nLC 1000 UHPLC system (Thermo Scientific). Peptides (2 μg) were injected onto an Acclaim PepMap100 C18 column (75 μm i.d. × 2 cm nanoviper, 3 μm particle size, 100 Å pore size) (Thermo Scientific) and further separated on a Acclaim PepMap100 C18 analytical column (75 μm i.d. × 50 cm nanoviper, 2 μm particle size, 100 Å pore size) (Thermo Scientific). The following 120 min method was used at 300 nl/min flow rate: starting with 100% buffer A (0.1% formic acid) with an increase to 5% buffer B (100% acetonitrile, 0.1% formic acid) in 2 min, followed by an increase to 35% buffer B over 98 min and a rapid increase to 100% buffer B in 6 min, where it was subsequently held for 10 min followed by equilibration with buffer A. The peptides eluting from the column were ionized by a nanospray ESI ion source (Thermo Scientific) and analyzed on the Q Exactive operating in positive-ion mode using electrospray voltage 1.9 kV and HCD fragmentation. Each MS/MS scan was acquired at a resolution of 35 000 FWHM, normalized collision energy (NCE) 28, automatic gain control (AGC) target value of 2 × 105, maximum injection time (mIT) of 120 ms and isolation window 2 *m/z*.

The specific peptides included for protein quantitation were: mAID (YISDWDLDPGR; VTWFTSWSPCYDCAR), mUNG (VEQNEQGSPLSAEQLVR; NVPAGFGESWK; LMGFVAEER), and mGAPDH (PITIFQER; GAAQNIIPASTGAAK; VPTPNVSVVDLTCR; LISWYDNEYGYSNR). Peptide areas for all the selected peptides (two to four) of the same protein were summarized to assign relative abundance to that protein. GAPDH levels were used for data normalization.

### Gene expression assay

Total RNA for mRNA analysis was prepared from CH12F3 cells harvested at several time points after seeding/stimulation (+CIT) using the mirVana miRNA isolation kit (Ambion) according to the manufacturer's instructions. RNA concentration and quality was measured on a NanoDrop ND-1000 UV-Vis spectrophotometer. Total RNA (770 ng) was reverse transcribed for gene expression analysis using TaqMan reverse transcription reagents (Applied Biosystems). The following TaqMan gene expression assays (Applied Biosystems) were used: *Aicda* (Mm01184115_m1), *Gapdh* (Mm99999915_g1), *Ung* (Mm00449156_m1). *Ung1* and *Ung2* specific gene expression was quantified with isoform specific customized TaqMan primers and probes, *Ung1* (Forw: CTGCTCGGCTGGACCAT, Rev: GCGCCAACCGCAAAGAC, Probe: CCGCCCCAAGACGC) and *Ung2* (Forw: AGTGGCGGCCGAGATC, Rev: CCACCCGGGCCTTCTTG, Probe: ATGCCGTGGCCAGCC). Quantitative reverse transcriptase PCR (qRT-PCR) was carried out on a Chromo4 (BioRad) real-time PCR detection system. Relative expression of mRNA was calculated by the ΔCt method using unstimulated cells at each time point as reference and *Gapdh* as control gene.

### Quantification of uracil in DNA by LC/MS/MS

Genomic uracil was quantified by a method described previously (29), but with some modifications. Cells (5–10 mill) were suspended in a buffer containing 10 mM Tris–HCl (pH 8.0), 10 mM NaCl, 1% SDS, 25 mM DTT, 0.1 mg/ml proteinase K (Worthington Biochemical), 0.1 mg/ml RNase A (Sigma-Aldrich), 50 μM deaminase inhibitor tetrahydrouridine (THU, Merck Millipore) and lysed by passing through 21G and 23G syringe needles followed by incubation at 37 °C for 1 h with 1000 RPM shaking. Proteins and lipids were subsequently extracted from the lysates with phenol:chloroform:isoamyl alcohol (25:24:1, Sigma), followed by two rounds with chloroform:isoamyl alcohol (24:1, Sigma). DNA (nuclear and mitochondrial) in the aqueous phase was precipitated by adding 0.3 volumes 10 M ammonium acetate (pH 7.9) and 1 volume 100% isopropanol. Pellets were washed twice in 70% ethanol. Residual RNA and free nucleotides were removed from the DNA samples by treatment with 50 μg RNaseA in 10 mM ammonium bicarbonate (pH 7.0)/10 mM MgCl_2_ for 30 min at 37 °C, followed by a subsequent isopropanol/ammonium acetate precipitation. The DNA pellets were washed twice in 70% ethanol, dissolved in water and the outputs were quantified. UNG-treated genomic DNA was used as a negative control. For these samples, 15 μg pooled genomic DNA from various mouse and human cells was treated with 77 ng of a truncated form of human UNG containing only the catalytic domain (Δ84UNG (21)) in 50 mM tris-HCl (pH 8.0), 1 mM EDTA, 0.5 mg/ml BSA, 1 mM DTT, 200 μM THU, and 8 U HindIII for 1 h, followed by isopropanol/ammonium acetate precipitation.

DNA (5–10 μg) was hydrolyzed to nucleosides by treatment with 0.8 U Nuclease P1 (Sigma-Aldrich), 80 U Benzonase (Santa Cruz Biotechnology), and 7.5 U Antarctic Phosphatase (New England Biolabs) in 50 μl reactions, containing 10 mM ammonium acetate (pH 5.5), 1 mM MgCl_2_, 0.1 mM ZnCl_2_ and 240 μM THU, for 60 min at 37 °C. The hydrolysis reaction was spiked with a heavy isotope-labeled internal standard, ^13^C^15^N_2_-dU. Enzymes were then removed from the reactions by adding 3 volumes of ice-cold acetonitrile. The tubes were incubated on ice for 10 min before centrifugation (16 000 g, 30 min, 4 °C) and supernatants were transferred to new tubes and lyophilized until dry.

To separate dU from dC, the samples were dissolved in water and fractionated on an Agilent 1100 HPLC system (with a UV detector set to 260 nm to identify the canonical nucleosides) and a mixed mode Primesep 200 column (2.1 mm × 150 mm, 5 μm, SieLC) kept at 30 °C using a flow rate of 0.4 ml/min and water and acetonitrile as mobile phase, each containing 0.1% formic acid, as the mobile phase. The 12-min-long HPLC gradient was as follows: 5% acetonitrile for 30 s, ramp to 35% acetonitrile by 1.5–2.5 min, and return to 5% acetonitrile by 2.51 min. The dU-containing fractions were collected from 1.6 to 1.7 min and vacuum centrifuged until dry.

Samples were dissolved in water and analyzed by LC/MS/MS using a reverse phase column (2.1 mm × 150 mm, 1.8 μm, EclipsePlusC18 RRHD, Agilent Technologies) kept at 25 °C with a flow rate of 0.3 ml/min on a 1290 Infinity II HPLC coupled to a 6495 Triple Quadrupole mass spectrometer with an electrospray ion source (Agilent Technologies). Water and methanol were used as the mobile phase, each containing 0.1% formic acid. The 13-min-long HPLC gradient was as follows: 5% methanol for 3 min, ramp to 13% methanol by 3.5 min, ramp to 17% methanol by 5.5 min to 7 min, and return to 5% methanol by 8 min. Analysis was performed in positive ionization multiple reaction monitoring mode, using the mass transitions (229.08 to 113.0 Da) for dU and (232.08 to 116.0 Da) for ^13^C^15^N_2_-dU (internal standard).

### Plasmid constructs, transfection and confocal microscopy

Constructs encoding mUNG1-GFP, mUNG1-1-n-GFP, mUNG2-GFP, mUNG2-1-49-GFP, hUNG1-GFP, hUNG1-1-39-GFP, hUNG2-GFP mutants, YFP-RPA2, and Cherry-PCNA have been described previously (16,17,22). To generate mUNG1-CFP and mUNG2-YFP, mUNG1 and mUNG2 cDNA were cloned into the pECFP-N1 and pEYFP-N1 vectors (Clonetech), respectively. Point mutations were introduced in the RPA2–binding site of mUNG1-GFP and mUNG1-CFP using the Quick-change site-directed mutagenesis kit (Agilent) according to the protocol. To generate inducible mUNG1-GFP and mUNG2-YFP, we used the tetracycline-inducible vector pCW57.1 (Addgene). Cells were transfected with FuGENE HD or X-tremeGENE HP (R oche) and analyzed 24 h post transfection. CH12F3 cells were transfected using Cell Line Nucleofector™ Kit L in Amaxa Nucleofector II device (Lonza) according to the manufacturer's instructions. Stably transfected cell lines were obtained by hygromycin selection (1.0 mg/ml) and YFP or GFP positive cells were sorted by a FACS Aria cell sorter. Cells were examined in a Zeiss LSM 510 laser scanning microscope (1 μm thickness) with a Plan-Apochromat 63×/1.4 oil immersion objective. CFP was excited at 458 nm and detected at 470–500 nm, YFP was excited at 514 nm with detection between 530 and 600 nm or excited with 488 nm and detected at 505–550 (when cotransfected with Cherry-PCNA), Cherry was excited at 543 nm and detected above 615 nm, and GFP was excited at 488 nm with detection between 505 and 550 nm.

### Statistical analysis

*P* values between two groups were calculated in Excel by the Student's *t*-test using two tailed distribution and two-sample equal variance.

## RESULTS

### Generation of UNG1 and UNG2 isoform-specific knockout clones in a mouse B cell lymphoma line

Due to the important role of UNG in Ig isotype switching, we decided to use the mouse B-cell lymphoma line CH12F3, which performs IgM to IgA switching after stimulation (30), as our primary cell model. Since the cellular localization of UNG isoforms has never been studied in B cells, we first generated stably transfected CH12F3 cells expressing tetracycline-inducible UNG1-GFP and UNG2-YFP. Using confocal microscopy of induced live cells, we verified that UNG1 and UNG2 were sorted mainly to the mitochondria and the nucleus, respectively (Figure 1B). However, in accordance with a previous study of mouse UNG1-GFP expressing cells (22), we also detected a faint UNG1-GFP signal in the nucleus (Figure 1B).

To investigate the roles of UNG1 and UNG2 separately, we generated isoform-specific knockout clones using CRISPR/Cas9 technology with single guide RNAs (sgRNAs) targeting *Ung1*- or *Ung2*-specific exon regions. In addition, we generated AID-knockouts as well as control cell clones by targeting intron 2 of the *Ung* gene (Table 1). After subcloning we screened and verified all knockout clones by western blot analysis (Figure 1C). Interestingly, UNG1 migrated as two protein bands, indicating post-translational modifications (PTMs, e.g. phosphorylation) or alternative proteolytic processing of the UNG1 N-terminus. Treatment of the cell extracts with phosphatase did not affect the migration patterns (Supplementary Figure S1). Thus, the two UNG1 variants likely represent unprocessed UNG1 (upper band) and MPP-processed UNG1 (lower band). We further verified the knockout clones by measuring UDG activity in whole cell extracts, using either a single stranded uracil-containing DNA oligo (Figure 1D) or double stranded U:A DNA as substrate (Figure 1E). The very low UDG activity when both UNG isoforms are lacking suggests that the contribution from the other UDGs was negligible on the uracil substrates tested. Finally, we identified the genetic editing events by deep sequencing and selected three unique clones to represent each knockout group (Table 2).

**Table 2.** CRISPR editing events in CH12F3 as revealed by deep sequencing

#	Subclone	Target residues	Indels	Mut	% of total reads
1	Ung1.4-B2	1 (M)	–10	-	91.1
2	Ung1.5-B2	13 (R)	–2	-	83.1
3	Ung1.5-B9	13 (R)/10 (R)	–2/–52	-	49.2/38.6
1	Ung2.1-A2	33 (A)/35 (E)	–10/–10	-	42.1/39.3
2	Ung2.1-A9	36 (I)	+1	-	78.2
3	Ung2.1-A28	35 (E)	–13	-	88.4
1	Ung1+2-A6	87 (F)/88 (G)	+32/–26	3/0	45.8/30.6
2	Ung1+2-B1	88 (G)/85 (A)	–10/+1	0/8	44.1/38.0
3	Ung1+2-B3	89 (E)	–2	-	85.8
1	Int2-A5	-	–4/–9	0/1	45.6/41.4
2	Int2-A6	-	0/–8	-	41.9/41.2
3	Int2-B1	-	–5/–4	1/1	45.5/39.9
1	Aicda-B11	23 (G)/23 (G)	–1/–5	-	48.7/46.2
2	Aicda-B39	23 (G)	–1	-	94.2
3	Aicda-B47	22 (K)	–3+1	2	94.3

Indels: Insertions (+) or deletions (–). Mut: mutations. Int2: Ung intron 2 (mock control). Differently edited alleles are separated by ‘/’.

### UNG1 and UNG2 are differentially regulated during the cell cycle in mouse B cells

**Figure 2.**
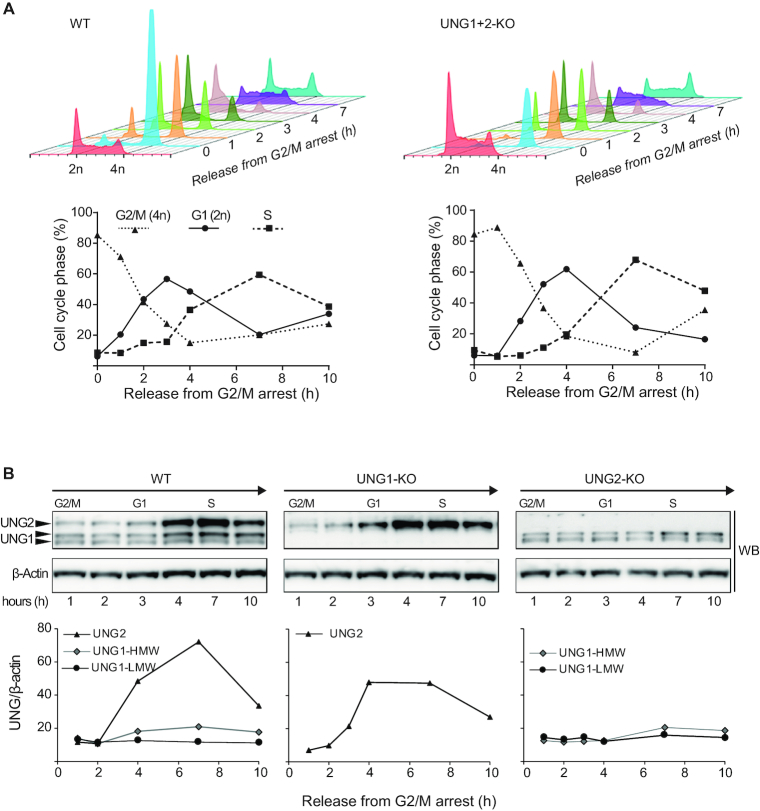
Cell cycle regulation of UNG1 and UNG2 in CH12F3 cells. (**A**) Cell cycle synchronization of WT and UNG1/UNG2-KO CH12F3 cells. Cells were arrested in G2/M using the ATP-competitive CDK1/cyclin B1 inhibitor RO-3306 and cell cycle distributions were analyzed by FACS at several time points after release (upper panels). For comparison, cell cycle histograms of unsynchronized cells are shown in red (in front). The fraction (%) of cells in each phase of the cell cycle at several time points after release from G2/M arrest is illustrated below the histograms. (**B**) Relative level of UNG1 and UNG2 isoforms during the cell cycle. Cells were harvested at several time points after release from G2/M arrest. UNG isoforms in WT and isoform-specific KO cells were analyzed by western blot on whole cell extracts (upper panel) and quantified relative to β-actin (lower panels). The two UNG1 variants (UNG1-HMW and UNG1-LMW) were quantified separately.

UNG is involved in removal of incorporated uracil at the replication fork during S-phase. We therfore investigated whether knocking out UNG (both isoforms) affected the cell cycle of highly-proliferating B cells. Cells were arrested at the G2/M transition by inhibiting CDK1/cyclin B1, and the fractions of cells in G2/M, G1, and S were measured by flow cytometry at several time points after release. We observed no difference in either freely-cycling or synchronized cells, demonsting that UNG deficiency has no significant impact on the cell cycle regulation of proliferating CH12F3 B cells (Figure 2A).

The expression patterns of UNG1 and UNG2 during the cell cycle have previously only been investigated in human non-lymphoid cell lines (31–33). However, the mouse *Ung* promoter regions show limited homology to the human *UNG* promoters (22), suggesting that regulation of UNG1 and UNG2 expression may be different in mouse cells. We therefore investigated the expression patterns of the UNG isoforms during the cell cycle in the CH12F3 mouse B cell line. Western blot analysis of G2/M-synchronized WT and isoform-specific knockout cells at several time points after release, showed that UNG2 is several-fold upregulated during the S-phase, while the expression of UNG1 (both forms) was almost cell cycle independent (Figure 2B). These results are in accordance with regulation of UNG isoform expression in human non-lymphoid cells (31–33), and demonstrate that cell cycle regulation of the UNG isoforms are independent of cell type and conserved between human and mice.

We next asked whether knocking out a single isoform affected the expression pattern of the other by quantifying UNG during the cell cycle in the isoform-specific knockouts. The knock-out of either isoform did not affect the other (Figure 2B). Taken together, we conclude that our mouse B cell isoform-specific UNG knockouts constitute a good model to investigate the individual biological function of each UNG isoform.

### Stimulation of CH12F3 cells has no significant impact on UNG expression

**Figure 3.**
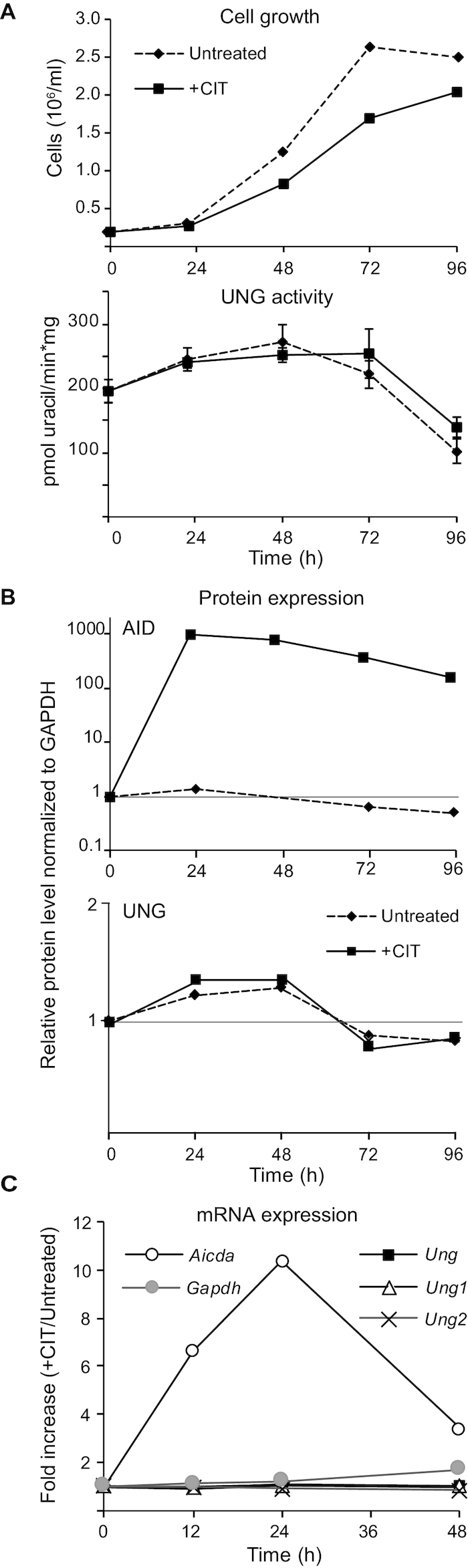
UNG expression in CIT-stimulated and unstimulated CH12F3 cells. (**A**) Cell growth (upper panel) and UDG activity (lower panel) in untreated and stimulated (+CIT) CH12F3 cells (WT). 200 000 cells/ml were seeded and samples were harvested for cell counting and UDG activity assays at several time points (0–96 h). UDG activity values represent the mean of five replicates at each time point. Standard errors are indicated on the curves. (**B**) Detection and relative quantitation of AID and UNG in untreated and stimulated (+CIT) CH12F3 cells cultures. Cells were seeded and stimulated as in A. At each time point AID (upper panel) and total UNG (lower panel) were quantified by targeted mass spectrometry. The data represent the mean of two biological replicates. All data are normalized to the protein level at the time of seeding (0 h). (**C**) *Ung1* and *Ung2* gene expression measured by RT-qPCR. The curves represent fold changes in gene expression in stimulated (+CIT) versus untreated cell cultures (+CIT/untreated). Values were calculated by the ΔCt method using untreated cells at each time point as reference. Each value represents the mean of two technical replicates. *Aicda* and *Gapdh* were included as positive and negative control genes, respectively.

UNG expression and activity are strongly induced in stimulated primary splenic mouse B-cells (10,34), and this has been reported to also occur in CH12F3 cells upon stimulation with anti-CD40, IL4 and TGF-β (CIT) (34). Newly isolated splenic B cells are mostly in resting phase and begin to proliferate when stimulated *ex vivo*. Thus, it is unknown whether this UNG induction is part of the immune response per se or due to increased proliferation. To address this question and to further characterize our cell model, we measured cell proliferation and UNG expression in CSR-stimulated and unstimulated CH12F3 cells. In contrast to primary cells, which are dependent on stimulation to proliferate, we observed a slightly reduced growth rate in CH12F3 upon stimulation (+CIT) (Figure 3A). Interestingly, UDG (UNG) activity did not increase in stimulated cells (Figure 3A). This was further verified at the protein level by quantitative MS. As expected, AID protein was strongly induced upon stimulation, while the level of UNG did not change (Figure 3B). Our quantitative MS method did not discriminate between the UNG isoforms. We therefore investigated the individual expression levels of UNG1 and UNG2 using qRT-PCR with isoform-specific probes. In accordance with the measurements at the protein level, a several-fold increase in AID mRNA expression was observed upon stimulation, with the highest level at 24 h (Figure 3C). By contrast, neither UNG1 nor UNG2 mRNA expression were affected by stimulation. This was also verified using a standard assay, which measures total UNG mRNA expression (Figure 3C). Taken together, this demonstrates that CSR-stimulation (CIT) is not a regulator of UNG in CH12F3 cells, neither at the level of enzymatic activity, total protein, nor relative expression of the isoforms.

### The UNG1 isoform supports Ig class switching

**Figure 4.**
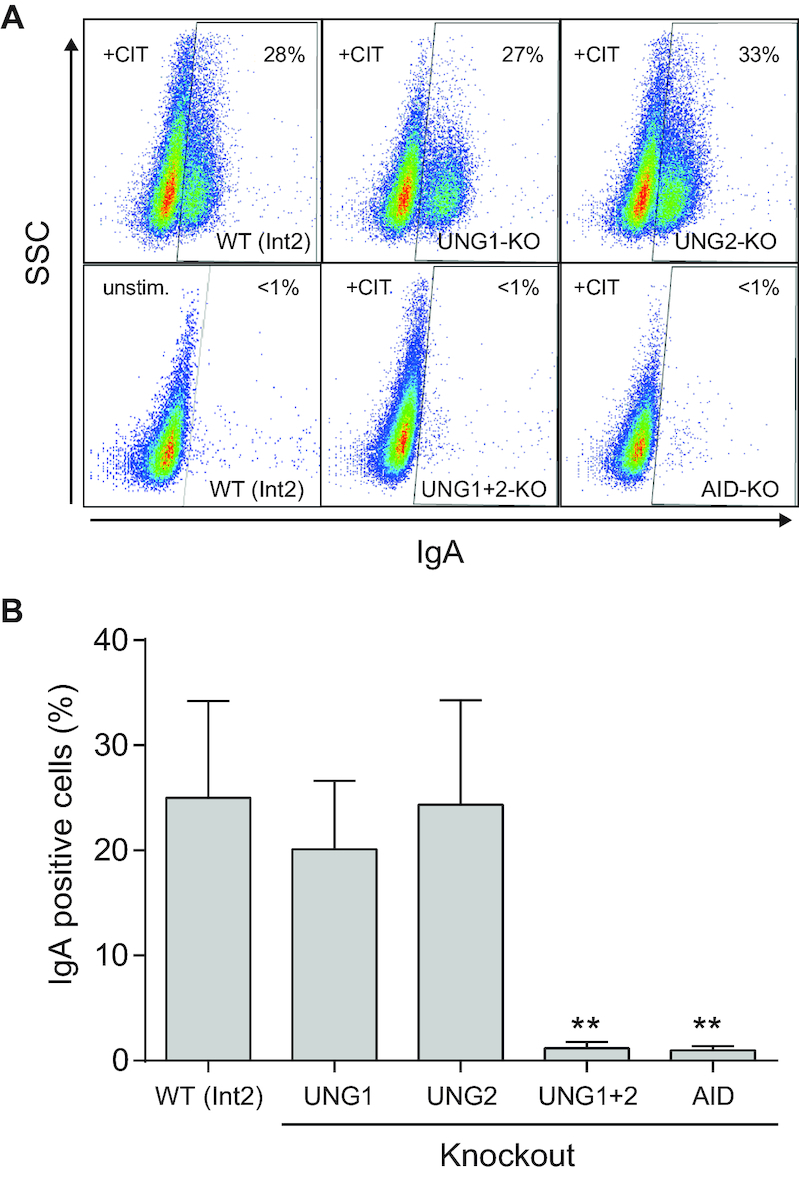
IgM to IgA class switching in isoform-specific UNG knockout clones. (**A**) Representative FACS analysis showing CSR to IgA in anti-CD40, IL4 and TGF-β (+CIT) stimulated CH12F3 WT (Int2), UNG1-KO, UNG2-KO, and UNG1/UNG2-KO cells. CH12F3 AID-KO and unstimulated WT (Int2) cells are included as negative controls. The fraction of IgA positive cells is indicated in the top left corner of each diagram. (**B**) IgM to IgA class switching activity in CH12F3 WT (Int2) and UNG-KO clones. The bars represent the mean switching activity (three biological replicates) of three independent clones in each group (see Table 2 for an overview of the clones). Significantly reduced CSR activity compared to WT (Int2) is marked with **P< 0.005.

The CH12F3 B-cell lymphoma cell line undergoes IgM to IgA switching *in vitro* after stimulation with CIT (30). It has generally been assumed that UNG2 is the UNG isoform responsible for processing of AID-induced uracil during CSR because of its nuclear localization, but this has not been specifically investigated. We therefore analyzed IgM to IgA switching in our panel of CH12F3 KO clones (Table 2). As expected, AID-KO and UNG1+2-KO cells were completely deficient in switching (Figure 4). By contrast, knocking out either UNG isoform had no impact on class switching efficiency (Figure 4). This unexpected UNG2-KO phenotype demonstrates that UNG1, as well as UNG2, can support CSR in murine B cells.

### UNG1 repairs genomic uracil in CH12F3 UNG2 knockout cells

**Figure 5.**
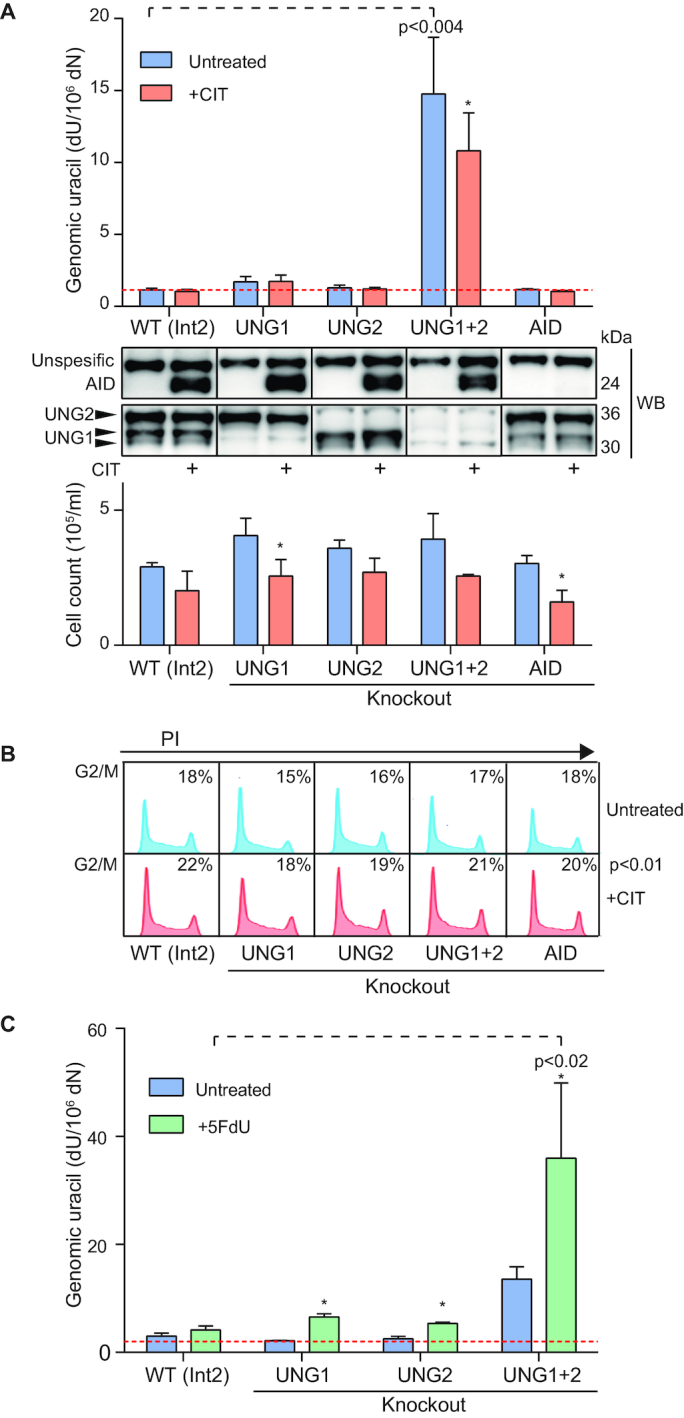
Genomic uracil levels in CH12F3 UNG isoform-specific knockout clones. (**A**) Genomic uracil (dU) levels in untreated and stimulated (+CIT) CH12F3 UNG isoform-specific knockout clones. All clones were seeded, cultured/stimulated, and analyzed in parallel. Uracil in DNA was quantified by LC–MS/MS. Each bar represents the mean value of three clones (see Table 2 for details). Red stippled line indicates dU level in UNG-treated DNA control (detection limit). Knockout clones with significantly different dU levels from WT (Int2) are indicated with *P* values. Significantly different dU levels in stimulated compared to untreated cells are indicated by asterisk (* indicates *P*<0.05). Western blots of AID and UNG were performed on each clone at harvest and representative clones are shown below the bar chart. The lower panel represents the number of cells at harvest (24 hours post seeding of 0.2 × 10^6^ cells/ml). Variation (standard error) between clones representing the same group is indicated by error bars. Significantly different cell growth in stimulated (+CIT) cells compared to untreated cells is indicated by asterisk. (**B**) FACS analysis of cell cycle distributions. Histograms of representative clones are shown. Harvested cells (same biological experiment as in A) were fixed and labelled with propidium iodide (PI). The fraction of cells in G2/M is indicated (-/+CIT). The numbers represent the mean of three different clones. Significantly higher fraction of cells in G2/M upon stimulation is indicated with *P* value. (**C**) dU levels in CH12F3 UNG isoform-specific knockout clones treated with thymidylate synthase inhibitor (5FdU). Cells were seeded 24 h prior to addition of 1 μM 5FdU and cultured (treated and untreated cells) for another 24 h before harvest. Uracil in DNA was quantified by LC–MS/MS. Bars represent the mean value of three different clones in each group. Red dotted line indicates dU level in UNG-treated DNA control (detection limit). 5FdU-treated clones with significantly increased dU levels compared to WT (Int2) or untreated cells are indicated by *P*-values or asterisk (*P< 0.05), respectively.

Based on the finding that UNG1 supports CSR of the *IgH* gene, we asked whether UNG1 also removes nuclear genomic uracil in general. To investigate this, we quantified total genomic uracil in all CH12F3 knockout clones listed in Table 2. Cells expressing only the UNG1 isoform (nuclear UNG2 knockout) as well as only the UNG2 isoform (UNG1 knockout) displayed similar genomic uracil levels as WT (WT and Int2 clones), while the UNG1+2 complete knockout clones had significantly higher levels of uracil in the genome (Figure 5A). These results demonstrate that the UNG1 isoform is sufficient to keep genomic uracil at WT levels in replicating cells. Thus, similarly to what we observed for CSR, UNG1 can also compensate for the lack of UNG2 during nuclear genomic uracil repair. Importantly, since our DNA samples contain nuclear as well as mitochondrial DNA (total genome), the results also show that lack of uracil repair in the mitochondria (UNG1 knockout cells) is not detected as an increase in the total genomic uracil level. This likely reflects the small amount of mitochondrial DNA relative to nuclear DNA.

We and others have previously shown that cancer cell lines constitutively overexpressing AID display increased levels of genomic uracil (34,35). We hypothesized that AID-induced genomic uracil may be repaired differently by the UNG isoforms and measured uracil levels in our isoform-specific knockouts after CIT stimulation. In contrast to previously published data on cells that constitutively overexpress AID (34,35), we did not observe any increase in total genomic uracil in CH12F3 cells stimulated by CIT to induce AID expression (Figure 5A). Neither did we observe reduced uracil levels in stimulated AID-KO cells compared to WT cells. Strikingly, for the UNG1+2 knockout clones, CIT-stimulation (AID induction) resulted in a slight but significant reduction in DNA uracil content. This was the opposite of what we expected and demonstrates that AID is not a major source of genomic uracil in CH12F3 cells. Importantly, this observation does not preclude the presence of a local AID-induced increase in uracil in *IgH* switch regions. However, similarly to the undetected but possible higher mitochondrial uracil-DNA levels in UNG1-KO cells, AID deamination does not contribute to a detectable increase in the total global uracil level. Genomic uracil levels may instead be dominated by replicative uracil incorporation, which is dependent on cell proliferation. Cell proliferation was actually slightly reduced by CIT-stimulation (Figures 4A and 5A). To explore this in more detail we performed cell cycle analysis. Upon stimulation, all genotypes displayed a similar pattern with a significantly higher fraction of cells in G2/M and lower fraction of cells in G1/S (Figure 5B). A reduced fraction of cells in S-phase, during which cells may incorporate dUMP, may explain the lower levels of genomic uracil observed in stimulated CH12F3 UNG1+2 knockouts. This is also in accordance with the finding that the reduced proliferation rates and prolonged G2/M phases in stimulated cells (also measured in cultures stimulated for up to ten days) were genotype independent, suggesting that neither AID deamination nor uracil-processing capacity is involved in the stimulation-induced growth restriction we observed.

In a further attempt to discriminate between the nuclear roles of UNG1 and UNG2, we treated the cell clones with the thymidylate synthase inhibitor 5-fluoro-2′-deoxyuridine (5FdU) to increase dUMP incorporation (36). The treatment significantly increased the levels of genomic uracil, and as expected, this was most prominent in the UNG1+2 knockout clones (Figure 5C). However, no significant difference was observed between the UNG1 knockout clones and the UNG2 knockouts (Figure 5C). This demonstrates that UNG1, which lacks the binding site for PCNA that guides UNG2 to the replication machinery and the site of dUMP incorporation (19), can remove incorporated uracil in the nuclear genome apparently as efficiently as UNG2.

In summary, we find that UNG1 functionally overlaps with UNG2 in the repair of genomic uracil in mouse B cells, including removal of incorporated uracil. In addition, our results suggest that replicative misincorporation of dUMP is the major source of genomic uracil in proliferating CH12F3 cells.

### Unprocessed UNG1 isoform variant is present in the cell nucleus while mitochondrial UNG1 entry is coupled to N-terminal processing

**Figure 6.**
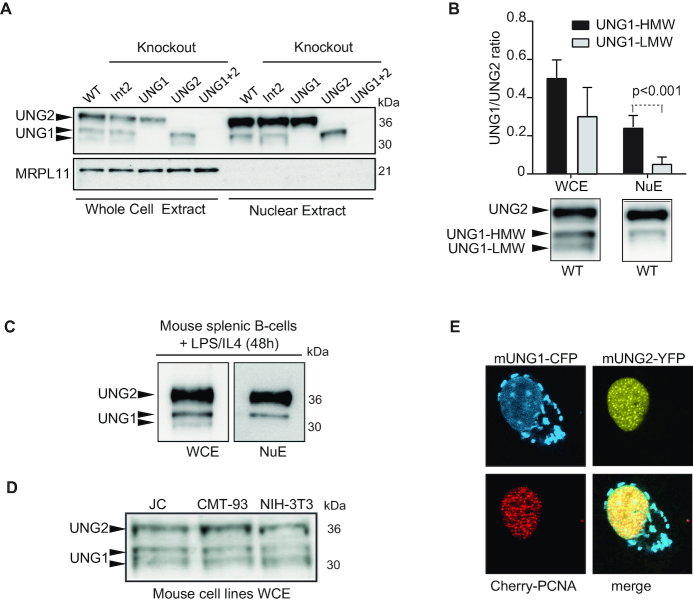
Identification of nuclear UNG1. (**A**) Western blot analysis of whole cell and nuclear extracts derived from CH12F3 clones. The mitochondrial protein MRPL11 (mitochondrial ribosomal protein L11, 39S subunit component) was used to validate the purity of the nuclear extracts. Equal amounts (total protein) are loaded in each lane. (**B**) Relative quantification of UNG isoform variants (UNG1/UNG2) in CH12F3 whole cell extracts (WCE) and nuclear extracts (NuE). The UNG1/UNG2 ratios in the different extracts from WT and Int2 clones were quantified from western blots. The bars represent the mean value of 5–6 biological replicates. Error bars are indicated. Two forms of UNG1 were observed in WCE, named UNG1-HMW (higher molecular weight) and UNG1-LMW (lower molecular weight). Western blots of UNG isoforms in WCE and nuclear extract derived from CH12F3 WT cells are shown below the bar diagram. (**C**) Naïve B cells were isolated from spleen, seeded (2 × 10^6^/ml), and stimulated with IL4 and LPS for 48 h before harvest and generation of whole cell extract (WCE) and nuclear extracts (NuE). UNG isoform variants were detected by western blot analysis. (**D**) Western blot analysis of UNG isoforms in several mouse cell lines (NIH3T3, JC and JMT-93). (**E**) Representative confocal image of live cells (HeLa) co-expressing mUNG1-CFP, mUNG2-YFP and Cherry-PCNA.

Our results demonstrate that endogenous UNG1 is active in the nucleus of CH12F3 cells, participating in class switching and genomic uracil repair. We observed that UNG1 migrates as two distinct bands on a western blot and asked whether the two bands represent mitochondrial and nuclear forms. Both variants were present in UNG2 knockout cells (Figures 1C, 2B, 6A, Supplementary Figure S3C). Using the isoform-specific clones, we isolated nuclear extracts and whole cell extracts in parallel and analyzed UNG by western blot. We observed that the nuclear extracts contained the highest molecular weight form of UNG1 (UNG1-HMW) in addition to UNG2 (Figure 6A). Quantification of western blots from several extract preparations revealed that UNG1-HMW was significantly enriched relative to the lowest molecular weight form of UNG1 (UNG1-LMW) in nuclear extracts, and UNG1-HMW constituted ∼25% of total nuclear UNG in WT cells (Figure 6B). To investigate whether the UNG1-HMW variant is present in primary cells, we isolated and stimulated naïve splenic B cells from mice and performed western blot analysis on whole cell and nuclear extracts. As with CH12F3 cells, nuclear extracts from primary mouse B cells contained the UNG1-HMW isoform together with UNG2 protein (Figure 6C). Moreover, we screened three non-lymphoid mouse cell lines and verified that the two UNG1 isoform variants were also present in cells of epithelial and fibroblast origin (Figure 6D). To characterize the UNG1 variants further, we isolated CH12F3 mitochondrial and cytosolic cell fractions (WT and UNG2-KO). When compared to WCE, we observed that UNG1 isoforms were enriched in the mitochondrial, but not cytosolic fraction (Supplementary Figure S3A, S3B, S3C). Moreover, we treated the intact mitochondria with Proteinase K (PK) to remove contaminants binding to the organelle surface. Only the processed variant (UNG1-LMW) displayed resistance to PK treatment, indicating that this form is contained inside the mitochondria and thereby shielded from PK degradation (Supplementary Figure S3C). In additon, we analyzed exogenously expressed UNG variants and used mouse UNG1 truncation mutants (mUNG1-1-n-GFP), together with the N-terminal fragment of human UNG1 (hUNG1-1-39-GFP) and the mUNG2-specific N-terminal residues (mUNG2-1-49-GFP) fused to GFP as controls (Supplementary Figure S2A). Human UNG1 is cleaved between residues 29 and 30 when entering the mitochondria (18), while UNG2 (human and mouse) does not undergo any known N-terminal proteolytic cleavage. We observed that mUNG1 N-terminal peptides mediated both nuclear and mitochondrial targeting (Supplementary Figure S2B). Interestingly, both mitochondrial targeting and N-terminal processing occurred in the NIH-3T3 mouse cell line (Supplementary Figure S2B, S2C), whereas neither were observed when mUNG1-1-38-GFP was expressed in the human U2OS cell line. Here mUNG1-1-38-GFP was detected as a single major band on the western blot and it localized to nucleus only (Supplementary Figure S2D). This was in contrast to the corresponding human construct (hUNG1-1-39-GFP), which displayed both efficient mitochondrial localization and N-terminal processing. Although is seems that there are some species differences between mouse and human UNG1, these results support that N-terminal processing is linked to mitochondrial targeting. To investigate this further, we fractionated mUNG1-1-38-GFP and GFP-expressing transfected NIH-3T3 cells into mitochondrial, nuclear and cytosolic fractions and analyzed the fractions by western blots using GFP antibody. GFP was present in all the cellular fractions at similar levels, while mUNG1-1-38-GFP was enriched in the nuclear and mitochondrial fractions (Supplementary Figure S3D). Importantly, the mitochondrial fraction contained mainly the processed (LMW) variant (Supplementary Figure S3D, S3E), while the unprocessed (HMW) variant dominated in the nuclear fraction (Supplementary Figure S3D). The UNG1-LMW variant was resistant to Proteinase K (PK) treatment (Supplementary Figure S3F), which further supports that this form of UNG1 is inside the mitochondria. We also performed MS analysis and identified peptides implicating that mUNG1-LMW is cleaved in the R18/S19/L20 region (Supplementary Figure S4), which fits with the observation that the processed form of mUNG1-1-38-GFP displayed a higher molecular weight than the processed form of its human counterpart (hUNG1-1-39-GFP), which is cleaved after residue 29 (Supplementary Figure S2C).

Finally, we compared subnuclear localization of UNG1 and UNG2 in cells expressing mouse UNG1-CFP together with mouse UNG2-YFP and Cherry-PCNA and observed that the nuclear localization patterns of UNG1 and UNG2 were different. Unlike the PCNA-binding UNG2 isoform, nuclear UNG1 displayed no preferential targeting to replication foci (Figure 6E). Thus, we found that mouse cells (cell lines and primary cells) express two forms of UNG1, of which the variant with the highest molecular weight is localized in the nucleus. The different subnuclear localization patterns of UNG1 and UNG2 indicate that they have specialized functions in the nucleus in addition to their overlapping roles in nuclear genomic uracil repair.

### UNG1 processes nuclear genomic uracil in human cells

**Figure 7.**
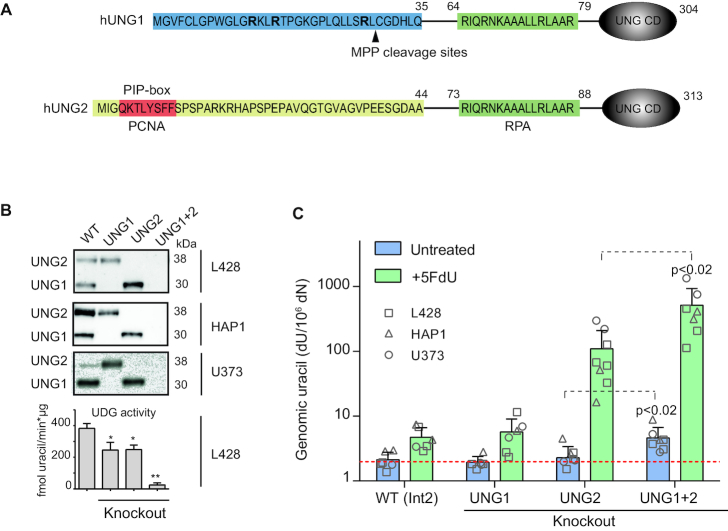
Generation and characterization of UNG isoform-specific human cell line clones. (**A**) N-terminal amino acid sequence of human UNG1 and UNG2. UNG1-specific residues (amino acid 1–35) are marked in blue. Arginine (R) residues in bold and target site for proteolytic processing by MPP (mitochondrial processing peptidase) are indicated. UNG2-specific residues (amino acid 1–44) are yellow, and include the PIP-box in red. The common RPA-binding site (green) and the globular catalytic domain (UNG CD) are indicated. (**B**) Representative CRISPR/Cas9 UNG isoform-specific knockout clones generated in three human cell lines (L428, HAP1, U373). Clones were screened by western blot analysis and UDG activity assays on whole cell extracts. High molecular weight ^3^H-U:A nick-translated DNA was used as substrate. The bars represent mean activity of three independent L428 clones. Significantly reduced UDG activity compared to WT (*UNG Int2*) is indicated with *P< 0.05 or **P< 0.005. (**C**) Genomic uracil levels in untreated and 5FdU-treated UNG isoform-specific knockout clones. Cells were seeded (0.2 × 10^−6^ cells/ml) 24 h before the addition of 1 μM 5FdU. Cells were harvested 24 h post treatment. dU was quantified by LC–MS/MS. Bars represent the mean value of six to eight different clones in each group (indicated). Red dotted line indicates dU level in UNG-treated DNA (detection limit). Significantly increased dU levels in the UNG1+2 knockout group compared to the UNG2 knockouts are indicated with *P*-values.

The specific N-terminal extensions of the UNG isoforms are not well conserved between human and mouse (Figures 1A and 7A). We therefore turned our attention to human cells and generated UNG isoform-specific knockout clones of two human cell lines of lymphoid origin (L428 and HAP1) and one glioblastoma cell line (U373), using the human single-guide oligo sequences listed in Table 1. We verified the clones by western blots and activity assays (Figure 7B), and the editing events were verified by deep sequencing (Table 3). Western blot analysis of human cell extracts displayed a single endogenous UNG1 band. However, exogenous overexpression of hUNG1-GFP and hUNG1-1-39-GFP demonstrated the presence of two UNG1 forms also in human cells (Supplementary Figures S2D and S5).

**Table 3.** CRISPR editing events in L428 as revealed by deep sequencing

#	Subclone	Target	Indels	Mut	% of total reads
1	UNG1-A3	17 (T)	–1/–5	1/0	55.8/32.7
2	UNG1-B6	17 (T)/18 (P)/15 (L)	–1/+1/–10	1/0/0	42.5/26.9/17.6
3	UNG1-B9	17 (T)/11 (L)/17 (T)	+19/–22/–9	1/0/0	36.2/24.4/12.9
1	UNG2-A3	1 (M)	–4/–3*	1/0	67.4/5.5
2	UNG2-A4	1 (M)	–14/–7/–10	3/0/0	22.8/22.7/21.5
3	UNG2-B1	1 (M)	–3*/–8	-	37.1/34.4
4	UNG2-B2	1 (M)	–6*/–12*/–1	1/0/0	23.9/23.1/21.7
1	UNG1+2-A2	90 (V)/92 (V)/88 (R)	–10/+1/–40	-	37.7/22.1/20.9
2	UNG1+2-A6	91 (P)/92 (V)	–5/+1	-	42.7/35.5
3	UNG1+2-B2	91 (P)	–1	-	82.7
1	Int2-A1	-	–34/–8/–5	-	29.5/28.1/27.6
2	Int2-A2	-	–8/–1	-	35.7/29.0

Indels: Insertions (+) or deletions (–). Mut: mutations. Int2: UNG intron 2 (mock control). Differently edited alleles are separated by ‘/’. (*) In-frame deletion accepted since start codon is destroyed. More than two unique editing events is due to partial polyploidy.

Genomic uracil (dU) levels were measured on 6–8 clones in each group. In untreated cells dU levels were significantly increased in the UNG1+2 knockout cells, but not in the UNG1- or UNG2 single-knockout cells (Figure 7C). This indicates that both isoforms can mediate nuclear uracil repair. However, UNG is a very efficient enzyme with high turnover number (9), so we conjectured that mitochrondrial UNG1 may deuracilate nuclear DNA during sample preparation. To test this, we added excess amounts of recombinant human UNG to cell lysates together with uracil-containing DNA oligo substrate, and performed a standard cleavage assay. UNG was completely inactive under lysis conditions (data not shown), demonstrating that the presence of UNG during cell lysis does not affect the quantitation of genomic uracil.

Finally, to try to discriminate between the human UNG isoform-specific knockout cells, we measured genomic uracil on cells treated with 5FdU. In accordance with the CH12F3 cell model, we measured similar levels of genomic uracil in human UNG1 knockout and WT clones (Figure 7C), indicating that mitochondrial uracil levels are either not increased by 5FdU treatment or mitochondrial DNA does not contribute significantly to total genomic uracil levels. By contrast, the 5FdU-treated human UNG2 knockout cells showed ∼20-fold higher uracil levels compared to WT, thus demonstrating that UNG2 plays a major role in genomic uracil-processing in human cells. However, 5FdU-treated UNG1+2 knockout cells showed a 5-fold increase in genomic uracil levels compared to UNG2 knockouts (100-fold increase compared to WT), which implies that UNG1 to some extent is capable of compensating for the loss of UNG2. All genotypes displayed similar growth rates and viability (∼95% live cells), so the higher genomic uracil levels were not due higher proliferative uracil incorporation. Thus, the increase in uracil levels in UNG1+2 knockout cells compared to UNG2 knockouts demonstrates that UNG1 performs nuclear uracil-processing in human cells.

## DISCUSSION

Two isoforms are expressed from the *UNG* gene: mitochondrial UNG1 and nuclear UNG2, each possessing unique N-terminal extensions that regulate cellular localization and protein interactions (13,15,17,19,31). To date, cell models to study the physiological function of each isoform separately have not been available. Here, we have generated UNG isoform-specific mouse and human cell models and investigated the regulation and function of endogenously expressed UNG1 and UNG2 separately. Overexpression of UNG proteins can lead to physiologically less relevant results (37–39), and it is preferable to use endogenously expressed variants that retain the expression patterns of WT proteins and do not affect overall cell cycle regulation. The cell lines we constructed fulfill these requirements. In mouse cells we identified a specific UNG1 isoform variant that is targeted to the cell nucleus where it supports Ig class switching and repairs genomic uracil. Moreover, by characterizing three human cell line models we demonstrated that UNG1 contributes to genomic uracil-processing also in human cells.

Mitochondrial targeting signal domains usually form amphiphilic helixes (40), while nuclear localization signals are typically composed of positively charged residue clusters (41). The N-terminal part of UNG1 posesses both of these features: it can form an amphiphilic helix (22) and it contains a high density of positively charged residues (Figures 1A, 7A and Supplementary Figure S2A). It can therefore act both as a mitochondrial targeting sequence and a nuclear localization signal. In accordance with this, the cellular localization of mUNG1 and mUNG1 N-terminal fragments fused to GFP displayed both nuclear and mitochondrial targeting (Figure 6E and Supplementary Figure S2). We observed two endogenous variants of mouse UNG1 with different molecular weights. The lowest molecular weight form of UNG1 constitutes the MPP-processed variant generated during mitochondrial entry, while the high-molecular-weight unprocessed form is in the nucleus (Supplementary Figures S2, S3).

By using the thymidylate synthase inhibitor 5FdU to increase the incorporation of dUMP, we show that the UNG1 isoform, efficiently removes incorporated uracil from the genome and apparently fully compensates for the absence of nuclear UNG2 in the mouse cells and partly so in human cells. Interestingly, 5FdU-treated human UNG1+2-KO cells displayed several-fold higher levels of genomic uracil compared to the corresponding mouse KO cells (Figures 5C and 7C, note log scale in 7C). This is likely because SMUG1 contributes relatively more to the total uracil-processing activity in mouse cells than in human cells (10,42), and may also explain why UNG1 only in part compensates for UNG2-deficiency in human cells. Since UNG1 does not bind to PCNA (Supplementary Figure S5), our results demonstrate that targeting of UNG to the replication machinery by PCNA is not essential for efficient uracil repair. Moreover, we demonstrate that direct UNG-PCNA interaction is not important for the role of UNG in CSR, as previously suggested (38).

Both UNG1 and UNG2 contain RPA binding sites (Figure 1). The interaction between UNG and RPA was originally detected using human UNG1 as bait in a two-hybrid screen in yeast (15). By pull-down experiments, cell imaging, and mutational analysis, we demonstrated that UNG1 actually interacts physically with RPA in the cell nucleus (Supplementary Figures S5 and S6). Trimeric RPA is a nuclear ssDNA-binding protein located at sites of DNA replication, repair, and transcription (43,44). Importantly, RPA is also central for the recruitment of AID to *Ig* loci during CSR and SHM (45,46). We have previously characterized a germline variant of the human UNG gene with abolished RPA binding (16). However, the variant allele was only detected in heterozygotes, and the functional relevance is therefore still unknown. It seems likely that RPA interaction is essential for the nuclear function of the UNG1 isoform variant identified here is likely, but this requires further investigation.

What is the evolutionary advantage of having two different UNG isoforms (UNG2 and UNG1-HMW) in the nucleus? There are numerous nuclear UNG forms due to more than 20 reported PTMs, including phosphorylation, acetylation and ubiquitination, with a majority of the PTM sites located in regions that are present in both UNG1 and UNG2 (www.phosphosite.org). The different modifications likely regulate protein turnover, enzymatic activity, localization and association with various proteins partners and DNA substrates (31,47), thus allowing fine-tuning of UNG protein function. UNG is unique among the glycosylases in that it can initiate both error-free genomic uracil processing by BER as well as mutagenic processing during antibody maturation in B cells (4). It removes newly incorporated uracil (U:A context) at the moving replication fork as well as deaminated cytosine (U:G context) in the overall genome. Moreover, it can efficiently excise uracil from ssDNA (9). In addition, UNG has been reported to have a function together with CENP-A at the centromere (48,49), and has been shown to promote TET-mediated DNA demethylation (50). Thus, the various UNG forms may reflect the involvement of this enzyme in these different nuclear processes.

**Figure 8.**
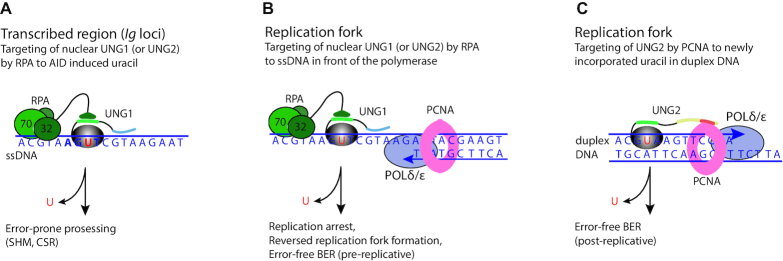
Simplified model showing targeting of nuclear UNG1 and UNG2 by RPA and PCNA to different genomic contexts. (**A**) Targeting of nuclear UNG1-HMW (or UNG2) to AID-induced uracil in transcribed ssDNA by the flexible winged helix (WH)-domain of RPA2 (32 kD). The positively charged N-terminus may interact with the negatively charged DNA backbone. UNG1 motifs are indicated with similar colors as in Figure 1A. The AID hotspot is indicated in bold. The action of UNG in this genomic context mediates error-prone processing during SHM and CSR. (**B**) Targeting of nuclear UNG1-HMW (or UNG2) by RPA to the ssDNA template strand in front of the moving replicative polymerase. The action of UNG1 (or UNG2) in this genomic context will result in replication arrest followed by formation of a reversed replication fork and pre-replicative error-free BER before the replication will then start up again. (**C**) Targeting of UNG2 by PCNA to newly incorporated uracil in duplex DNA behind the moving replicative polymerases (POLδ and POLϵ). UNG2 motifs are indicated with similar colors as in Figure 1A. The action of UNG2 in this genomic context will mediate post-replicative error-free BER.

It is plausible that the action of UNG is controlled by regulating its interaction with the hub proteins PCNA and RPA, which binds duplex DNA and ssDNA, respectively (Figure 8). The nuclear UNG1 variant, which lacks a PCNA binding site (Supplementary Figure S5), may be more dedicated to uracil in ssDNA, or alternatively to ssDNA-dsDNA junctions (20), through its interaction with RPA. In B cells such sites are present in RPA-stabilized ssDNA regions of the *Ig* loci that are targeted by AID (46) (Figure 8A). The action of UNG at these stable ssDNA regions may promote error-prone processing since conventional BER requires duplex DNA with a template strand. RPA may also guide UNG to the template strand directly in front of the replicating polymerase (Figure 8B). Single stranded DNA is ∼100-fold more prone to deamination than duplex DNA (1). Thus, it is critical for the cell to remove uracil in the template strand to prevent mutations. A scenario that can occur downstream of uracil-excision in this genomic context is replication arrest followed by formation of a reversed replication fork that can be used as substrate for error-free BER (Figure 8B).

UNG2 can bind both PCNA and RPA (Supplementary Figure S5). PCNA encircles duplex DNA and guides UNG2 to newly replicated DNA, thereby facilitating removal of incorporated uracil (19) (Figure 8C). The action of UNG2 in this genomic context will promote error-free BER by accessing the template strand. Whether UNG2 is targeted to ssDNA or duplex DNA is likely regulated by PTMs in the PCNA- and RPA-binding motifs. Interestingly, the most frequently reported PTM in UNG is phosphorylation at Tyr8 (www.phosphosite.org), which is located in the PIP-box (Figures 1A and 7A), and phosphorylation of this residue impede binding to PCNA (47). Likewise, there are several reports on ubiquitination of human UNG2 K78 (www.phosphosite.org), which is located in the RPA binding site (Figure 7A). Although not yet experimentally proven, one could easily imagine that a large ubiquitin group at this position would disrupt binding to RPA. Thus, phosphorylation and ubiquitination at the PCNA and RPA interaction motifs, respectively, may serve as a strategy to regulate targeting of UNG to different genomic contexts. However, an alternative and more direct strategy for differential targeting could be the engagement of nuclear UNG isoforms: the UNG1 variant with a single RPA-binding site and UNG2 with an additional PCNA-binding site. Further studies are therefore required to elucidate the fine-tuning of UNG targeting and repair, as well as its role in biology and disease.

## Supplementary Material

Supplementary Data
