# Supplementary material for: Uracil–DNA glycosylase UNG1 isoform variant supports class switch recombination and repairs nuclear genomic uracil
Source: Nucleic Acids Res. 2019 Mar 6;47(9):4569–85. doi: 10.1093/nar/gkz145 (PMC6511853; doi:10.1093/nar/gkz145)
Supplement: Supplementary Data [file gkz145_supplemental_file.pdf]

Supplementary

Uracil-DNA Glycosylase UNG1 Isoform Variant Supports Class Switch Recombination and Repairs Nuclear Genomic Uracil

Antonio Sarno[1, 2, 3], Marie Lundbæk[1], Nina Beate Liabakk[1], Per Arne Aas[1], Robin Mjelle[1], Lars Hagen[1, 3], Mirta M. L. Sousa[1, 2, 3], Hans E. Krokan[1] and Bodil Kavli[1, 2]*

[1] Department of Clinical and Molecular Medicine, NTNU-Norwegian University of Science and Technology, NO-7491,Trondheim, Norway.
[2] Clinic of Laboratory Medicine, St. Olav's Hospital, Trondheim University Hospital, NO-7006 Trondheim, Norway
[3] PROMEC Core Facility for Proteomics and Modomics at NTNU and the Central Norway Regional Health Authority

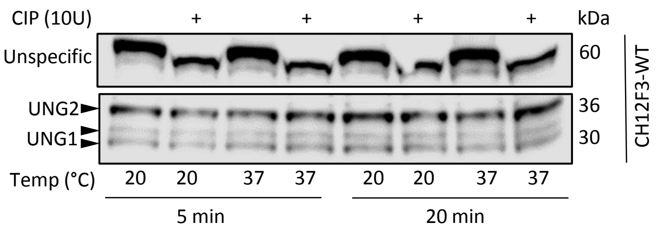

Figure S1. Phosphatase (CIP) treatment of CH12F3 cell extracts analyzed by western blot; no effect on UNG1 band migration. Whole cell extract were generated as described in the method section, except that phosphatase inhibitor cocktails were excluded. Extracts (100 µg total protein) were treated with CIP (10 U) in10 µl reactions at different time points and temperatures as indicated. Samples representing 50 µg protein were analyzed by western blot analysis using antibody against mouse UNG as described. An unspecific band (~60kD) on the same western blot indicated active CIP treatment but CIP did not change the migration pattern on the UNG1 protein bands.

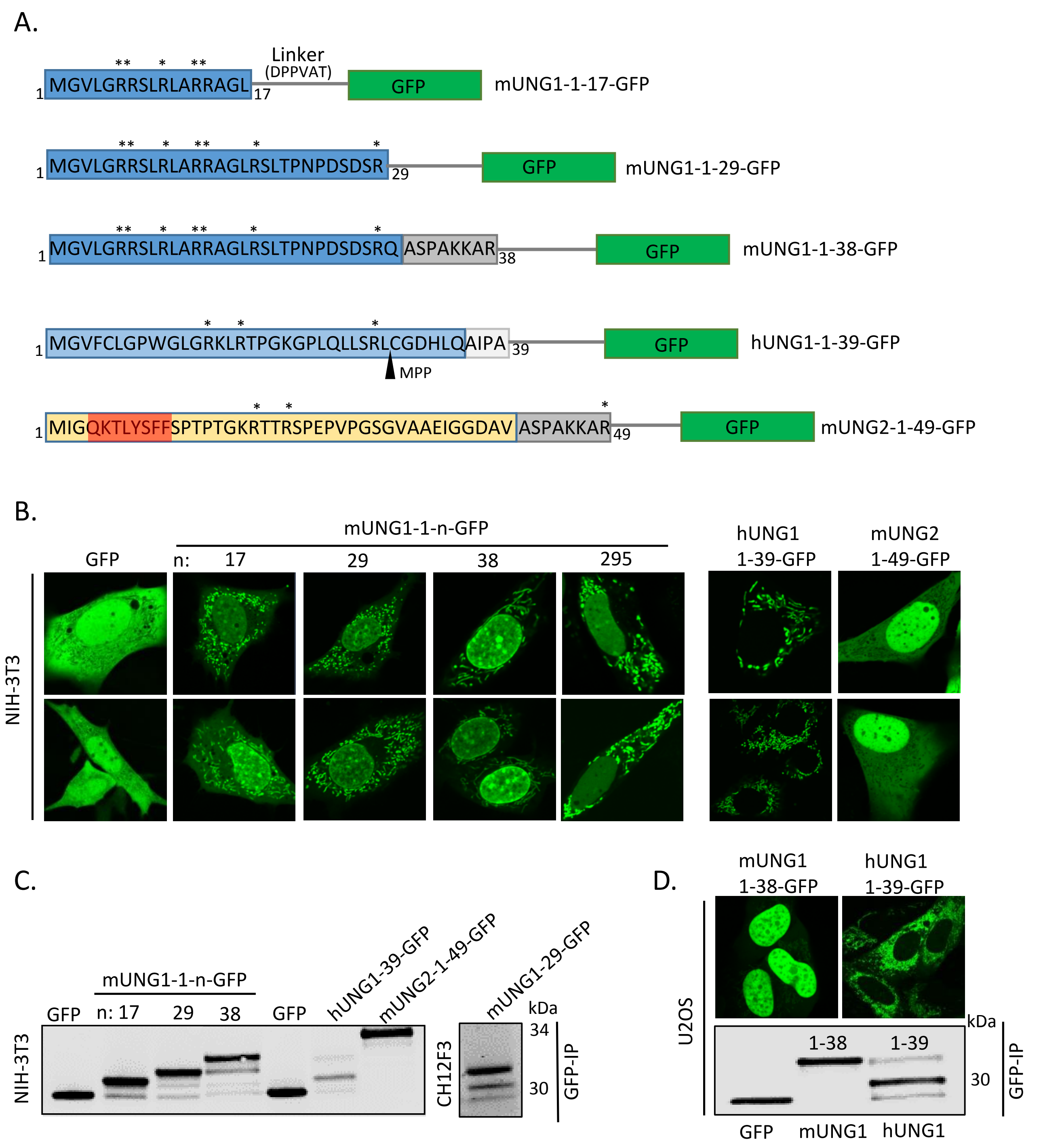

Figure S2. Cellular targeting and N-terminal processing of various exogenously expressed UNG-GFP variants; mUNG1 contains a combined nuclear and mitochondrial localization signal. A. Schematic illustration of the UNG-GFP constructs analyzed. UNG1 and UNG2-specific residues are marked in blue and yellow, respectively. Arg (R) residues are highlighted with asterisk (*) and residues encoded by the linker region are indicated. **B**. Confocal microscopy images of live NIH-3T3 cells expressing UNG-GFP constructs as indicated. **C**. Western blot analysis of GFP fusion proteins immune-precipitated from transfected NIH-3T3 or CH12F3 cells (200 µg WCE) using covalently coupled polyclonal GFP Ab (made in house) on protein A Dynabeads (Invitrogen). Proteins were detected with GFP Ab (ab 290) and IRDye 800CW Goat anti-rabbit. **D**. Confocal microscopy images of live U2OS cells expressing the N-terminus of mUNG1(1-38) and hUNG1(1-39) fused to GFP. Western blot of the proteins expressed in U2OS is shown below.

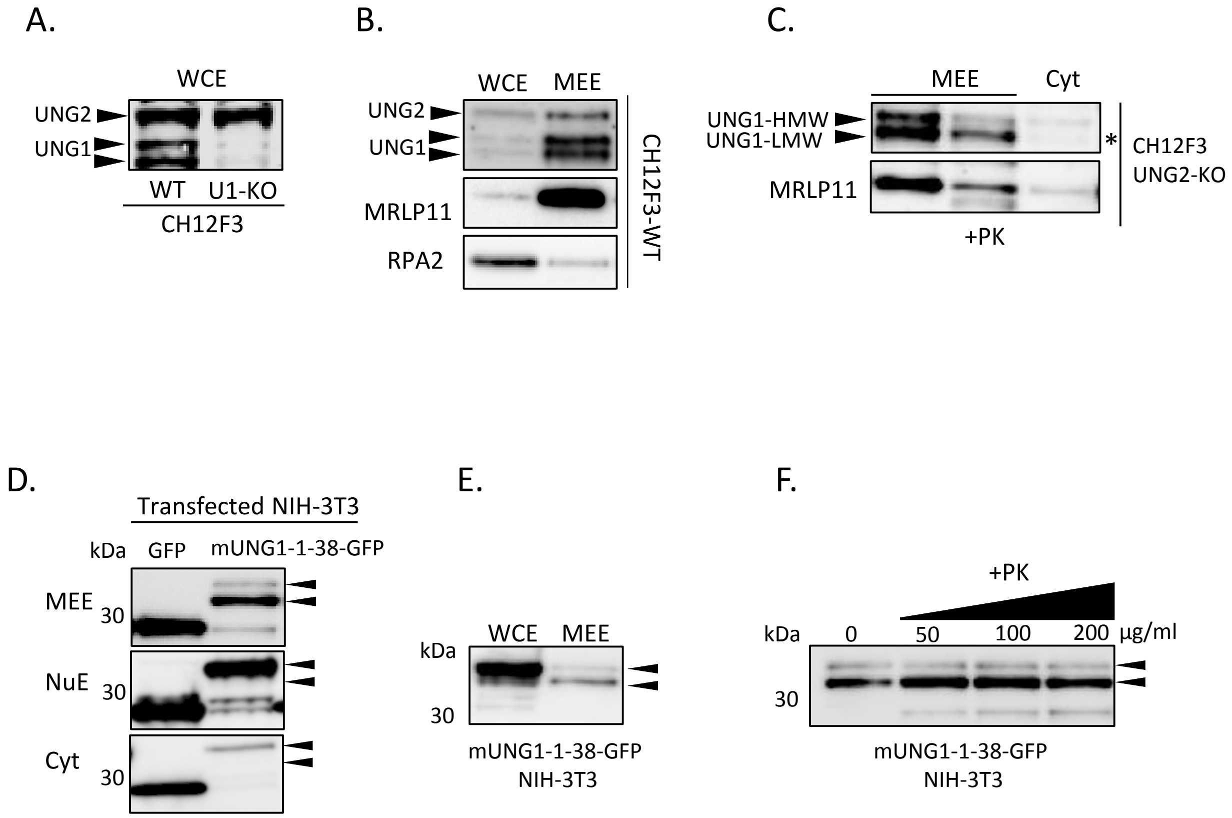

Figure S3. UNG1-LMW is the mitochondrial UNG1 variant. A. UNG western blot of 50 µg WCE derived from CH12F3 WT and UNG1-KO cells. **B.** Western blot comparing equal amounts (7 µg total protein) of WCE and mitochondria-enriched extract (MEE) derived from CH12F3 WT cells. The mitochondrial protein MRLP11 was used as mitochondrial marker and RPA2 was used as a nuclear marker. **C**. Mitochondria were isolated from CH12F3 UNG2-KO cells. Half of the mitochondria were treated with 60 µg/ml Proteinase K (PK) at 20°C for 30 min before preparation of mitochondrial extracts. Extracts generated from equal amounts of untreated and PK-treated mitochondria and the cytosol (Cyt) fractions were loaded on the gel. UNG1-HMW is degraded while the UNG1-LMW variant is more resistant to the PK treatment (marked with *). **D.** Western blot of transfected NIH-3T3 cells comparing GFP and mUNG1-1-38-GFP in mitochondrial (MEE), nuclear (NuE), and cytosolic fractions. Note that the processed form of mUNG1-1-38 dominates in MEE but is not detected in the NuE fraction **E**. Western blot showing UNG1-1-38-GFP in WCE compared to MEE. **F**. The processed form of mUNG1-1-38-GFP is resistant to PK treatment. Mitochondria were treated with increasing amounts of PK as indicated at 37°C for 30 min.

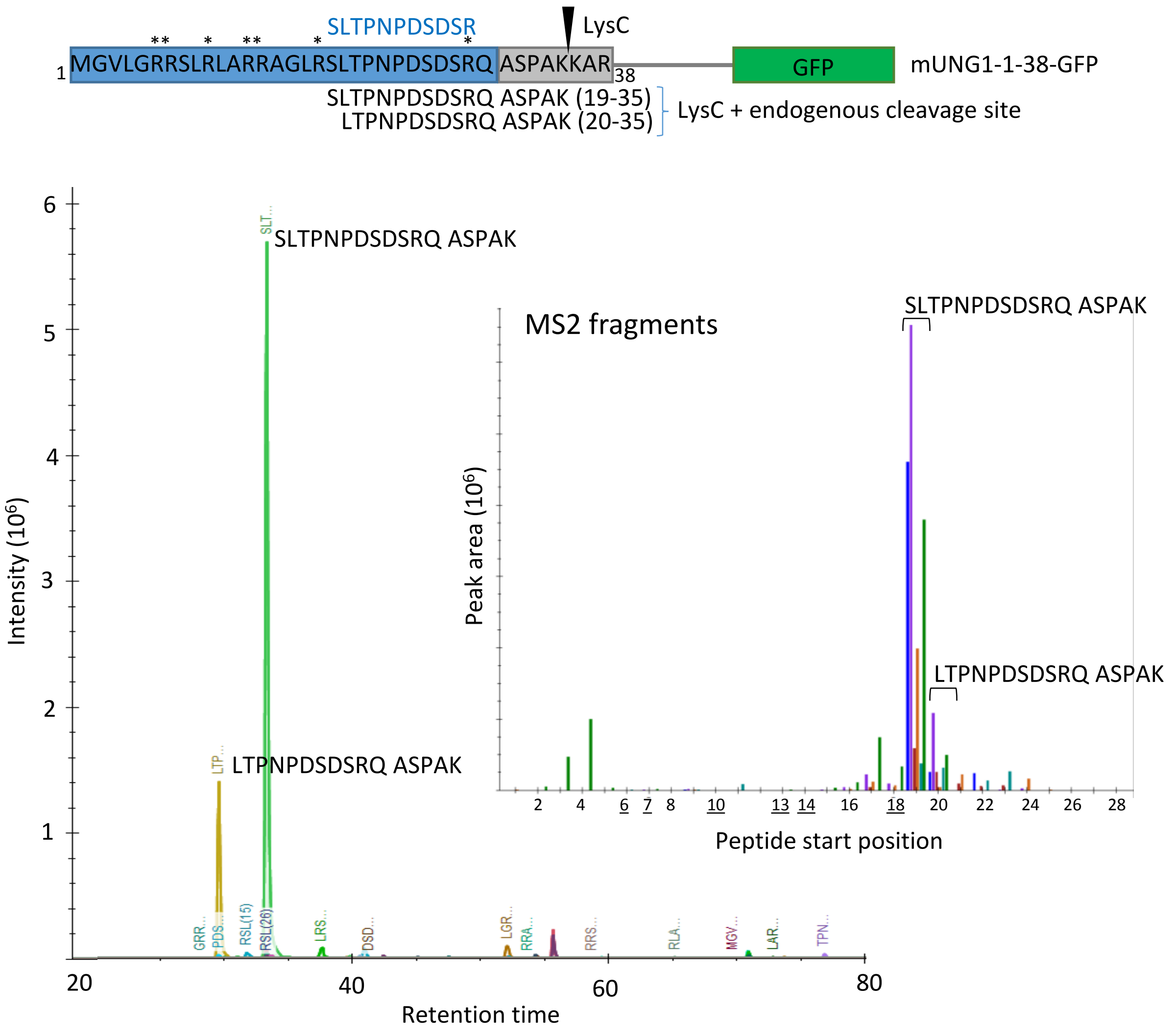

Figure S4. Identification of the proteolytic cleavage sites in mouse UNG1 N-terminal region by targeted MS. Endogenously expressed UNG1; peptide generated by trypsin in blue (mUNG1-19-29) was identified in CH12F2 UNG2-KO cell extracts. All other data are from exogenously expressed mUNG1-1-38-GFP. mUNG1-1-38-GFP was immuneprecipitated (GFP-IP) from transfected NIH-3T3 cells followed by LysC digestion (cleaves after K) on the beads. Further, all possible peptides after eventual proteolytic cleavage in the N-terminal region were targeted using PRM-based targeted mass spectrometry (see materials and methods section). Data analysis using Skyline (UW, Seattle, WA) identified peptides (SLTPNPDSDSRQ ASPAK/LTPNPDSDSRQ ASPAK), shown by peak intensity and peak area of the MS2 fragments, corresponding to proteolytic cleavage of mUNG1 between R18/S19 and S19/L20.

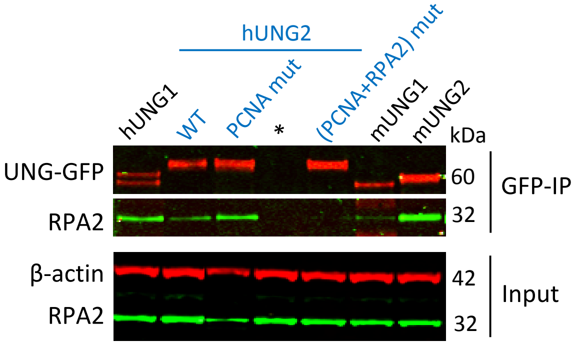

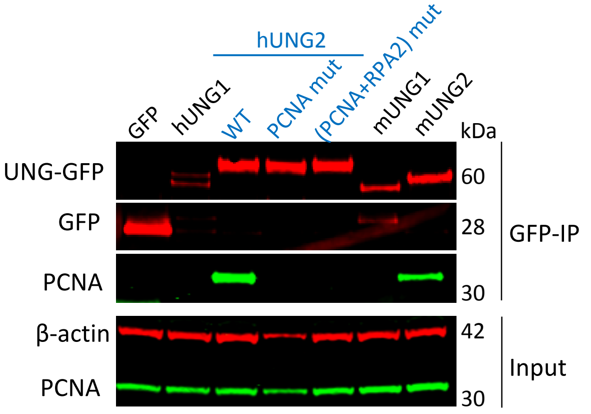

QKTLYSFF	PCNA binding site (PIP box)
QKTLYSAA	mutated PCNA binding site
RIQRNKAAALLRLAAR	RPA2 binding site
RIQRDKAAALLDLAAR	mutated RPA2 binding site

Figure S5. RPA2 and PCNA pull down experiments; UNG1 binds RPA and UNG2 binds RPA and PCNA. U2OS cells were transfected with various UNG-GFP constructs (h: human, m: mouse) as indicated and GFP-IP were performed from WCE (500 µg total protein) as described. Nuclease-treated extracts were used to pulldown PCNA. Western blot analysis were performed on IP and input. UNG-GFP variants (and GFP control) were detected by GFP Ab (ab 290) and IRDye 680RD goat anti-rabbit (red). RPA2 and PCNA were detected with primary antibodies RPA2 Ab [EPR2877Y] (ab76420) and PCNA Ab (ab18197), respectively, using IRDye 800CW Goat anti-rabbit as secondary Ab (green). β-actin (input loading control) was detected by monoclonal mouse anti-β-Actin (ab 8226) and IRDye 680RD goat anti-mouse (red). Proteins were visualized on a LI-COR Odyssey Imaging system. The mutations in the PCNA and RPA2 binding sites are indicated.

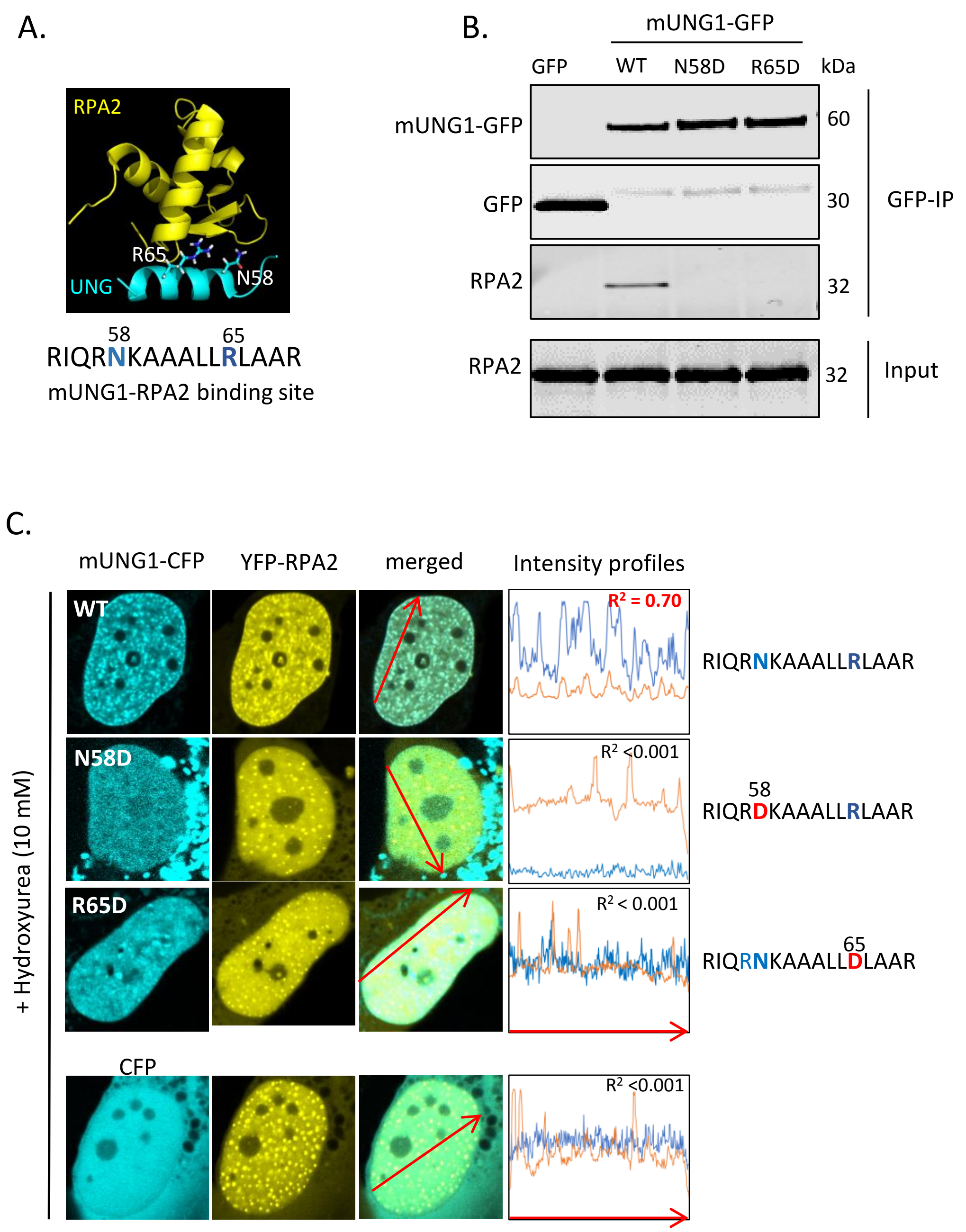

Figure S6. mUNG1 interacts physically with RPA in the nucleus. A. Ribbon structure of the C-terminal WH-domain of RPA2 bound to the UNG RPA binding motif. The figure was generated using PyMOL Molecular Graphic system and coordinates PDB ID 1DPU. The side chains representing mUNG1 N58 and R65 are visualized. **B.** RPA2 pull-down experiment (GFP-IP) were performed as described, using mUNG1-GFP (WT and mutants) as bait. WCE were generated from U2OS cells transfected with the constructs indicated. UNG1-GFP and RPA2 were detected as described in figure S4 **C.** Confocal microscopy images of live U2OS cells treated with hydroxyurea for 24 h. Cells were transfected with various mUNG1-GFP constructs as indicated. Colocalization of mUNG1 and RPA2 in the nucleus were analyzed using the LSM 510 META line profile software tool and regression analysis (R^2) of CFP and YFP intensity along the line (blue and yellow curves, respectively) were performed in Excel.
